# A simple, enaminone-based approach to some bicyclic pyridazinium tetrafluoroborates

**DOI:** 10.3762/bjoc.9.166

**Published:** 2013-07-23

**Authors:** František Josefík, Markéta Svobodová, Valerio Bertolasi, Petr Šimůnek

**Affiliations:** 1 Institute of Organic Chemistry and Technology, Faculty of Chemical Technology, University of Pardubice, Studentská 573, CZ 532 10, Pardubice, Czech Republic; 2 Dipartimento di Scienze Chimiche e Farmaceutiche, Centro di Strutturistica Diffratometrica, Università di Ferrara, Via L. Borsari 46, 441 00, Ferrara, Italy

**Keywords:** azo coupling, diazonium salt, enaminone, pyridazinium

## Abstract

Easily obtainable cyclic enaminones (piperidin-2-ylidenealkanones) can be transformed into substituted bicyclic pyridazinium tetrafluoroborates upon treatment with corresponding diazonium salts. The transformation can be performed either in a one-pot way or in a two-step process with the isolation of single azo-coupled enaminone as the intermediate. The former method is superior. Under the optimized conditions, a number of pyridazinium salts substituted with both electron-donating and electron-withdrawing substituents was easily synthesized. A mechanism of the formation of the pyridazinium salts is suggested. A partial drawback is the possibility of the formation of a mixture of products when using a different diazonium salt in each step due to a reversibility of the azo coupling. This can be suppressed by using a more reactive diazonium salt before a less reactive one.

## Introduction

**Scheme 1:**
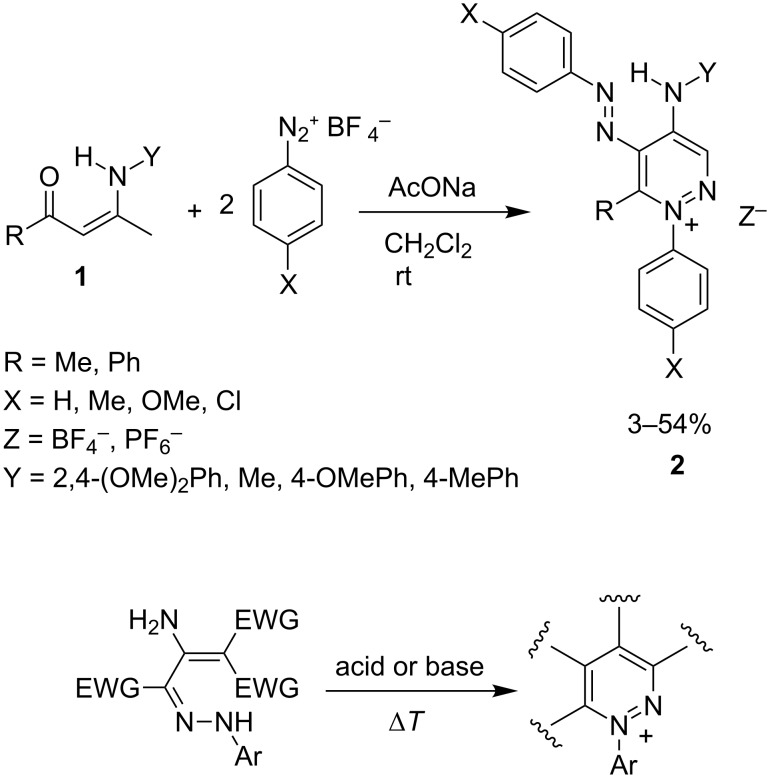
Syntheses of 1-arylpyridazinium salts.

As heterocyclic compounds play a very important role in everyday life, e.g., as pharmaceuticals, agrochemicals, dyes, etc. (for many monographies or textbooks on this topic see, e.g. [1]), searching for new heterocyclic systems or novel methods for the synthesis of the existing ones represents a very important part of organic chemistry. Enaminones, due to their ability to react with both electrophiles and nucleophiles, are versatile synthons, and not only in heterocyclic chemistry (for many reviews see, e.g., [2–6]). During the past several years we have dealt with the reaction of various enaminones with diazonium salts and established a number of methods for the construction of some important or unusual heterocyclic systems [7–15], including a simple protocol leading from enaminones **1** to 6-substituted 4-amino-5-aryldiazenyl-1-arylpyridazinium salts **2** [14–15]. The procedure consists of the reaction of the corresponding enaminones and two equivalents of diazonium tetrafluoroborates or hexafluorophosphates with the later giving better yields and applicability (Scheme 1, top).

It extends the existing procedure [16–24] for the construction of the 1-arylpyridazinium scaffold based on acid- or base-catalyzed cyclization of the corresponding hydrazones (Scheme 1, bottom). Apart from this, reactions of enaminones with diazonium salts giving substituted pyridazines are also described [25–28]. Our method does not require any additive, and the transformation from **1** to **2** is performed as a one-pot procedure. Different substrates are used and different and novel pyridazinium patterns are built under mild conditions [14–15].

**Scheme 2:**
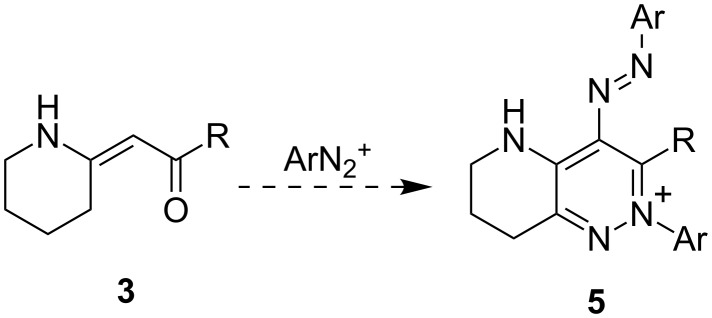
Suggested transformation of the cyclic enaminones into the corresponding bicyclic pyridazinium salts.

The above-mentioned method [14–15] (Scheme 1, top) is, however, limited to electron-donating or slightly electron-withdrawing (-Cl) substituents on diazonium salts. Only the enaminones having a C=C(NHR)–CH_3_ fragment were successfully applied. In this study we decided to further examine the reaction of enaminones with diazonium tetrafluoroborates in order to extend the applicability of the method. As the substrates, we have chosen the exocyclic enaminones **3**, which would gain access to bicyclic pyridazinium salts **5** (Scheme 2).

## Results and Discussion

**Scheme 3:**
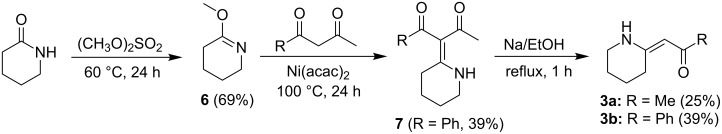
The synthesis of the starting β-enaminones.

Enaminones **3** were prepared as previously reported [12] (Scheme 3). The last step of the synthesis is the deacetylation of **7** by sodium in ethanol. In the case of substrate **3a** the deacetylation spontaneously takes place during the 2nd step.

The reaction of the enaminone **3a** with two equivalents of 4-methylbenzenediazonium tetrafluoroborate under the same conditions as described in [8] (i.e., DCM, rt, 2 equiv of diazonium salt, 6 equiv of sodium acetate) afforded the corresponding pyridazinium salt **5a** in 36% yield. The same procedure applied to the benzoylacetone derivative **3b** gave even lower yield (19%) of the salt **5g**.

**Scheme 4:**
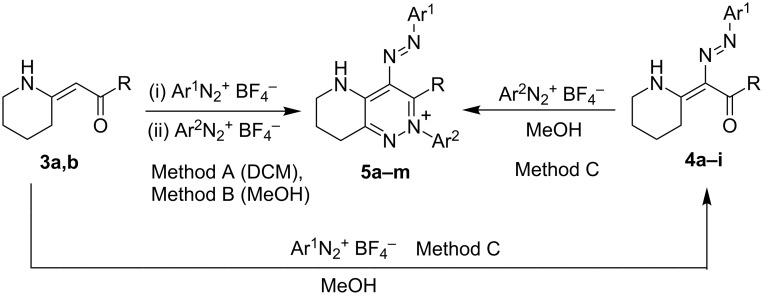
Synthesis of the bicyclic pyridazinium salts using different methods.

The cause of the low yields probably lies in the method of performing the reaction. The solid diazonium salt, added in one portion, could partially decompose before being able to react causing both lowering of the yield and contamination of the reaction mixture with the decomposition products. Therefore, the first attempt to improve the yields (method A) consisted in portionwise addition of the diazonium salt. The second portion was added after the first one had been fully consumed (tested by chromotropic acid). Using this method, we were able to prepare also mixed pyridazinium salts from two different diazonium salts (Scheme 4, Ar^1^ ≠ Ar^2^). However, the yields were still, at best, moderate (Table 1).

**Table 1:** Comparison of the individual methods for the synthesis of the salts **5**.

Entry	Salt	R	Ar^1^	Ar^2^	Method A^a^ Yield [%]	Method B Yield [%]	Method C^b^ Yield [%]

1	**5a**	Me	4-MePh	4-MePh	40 (12)	70	41 (**4a** 89)
2	**5b**	Me	4-MeOPh	4-MeOPh	39 (20)	92	81 (**4b** 84)
3	**5c**	Me	4-BrPh	4-BrPh	47 (20)	60	61 (**4c** 78)
4	**5d**	Me	4-Et_2_NPh	4-Et_2_NPh	24 (10)	45	50 (**4d** 76)
5	**5e**	Me	4-NO_2_Ph	4-NO_2_Ph	24 (5)	57	79 (**4e** 88)
6	**5f**	Me	4-NO_2_Ph	4-Et_2_NPh	16	85	85 (**4e** 88)
7	**5g**	Ph	4-MePh	4-MePh	50 (10)	80	71 (**4f** 95)
8	**5h**	Ph	4-MeOPh	4-MeOPh	10 (20)	72	74 (**4g** 90)
9	**5i**	Ph	4-BrPh	4-BrPh	40 (15)	53	55 (**4h** 58)
10	**5j**	Ph	4-Et_2_NPh	4-Et_2_NPh	32 (12)	26	^c,^ (^d^)
11	**5k**	Ph	4-NO_2_Ph	4-NO_2_Ph	1 (0)	61	68 (**4j** 93)
12	**5l**	Ph	4-MePh	4-Et_2_NPh	15	93	73 (**4f** 95)
13	**5m**	Ph	4-NO_2_Ph	4-MeOPh	–^e^	76	85 (**4j** 93)

^a^Yields for 6 equiv of AcONa are in parentheses. ^b^Yield for the 2nd step. Yields for the 1st step (product **4**) are in parentheses. ^c^Failed. ^d^Product **4i** is too unstable to be isolated and was used directly in the next reaction step. ^e^Not performed.

Method A was then modified. To achieve better homogeneity of the reaction mixture, a solution of the diazonium salt in methanol was used instead of the solid salt (method B). This modification led to a substantial increase of the yields (Table 1).

Further modification (method C) comprises step-by-step implementation of the methodology B. After the consumption of the first equivalent of the diazonium salt the reaction mixture was worked-up and the product was isolated and characterized by means of ^1^H and ^13^C NMR in solution and elemental analysis or HRMS. The isolated intermediates **4** were subjected to the reaction with another equivalent of the diazonium salt. The same products as in the case of the one-pot procedures (methods A,B) were isolated (Scheme 4). For comparison of the stepwise procedure (method C) with the other ones see Table 1. The methodology failed only in the case of compound **4i** (Table 1, entry 10), which was too unstable and decomposed upon an attempt of its isolation.

The pyridazinium salts prepared were characterized by means of ^1^H and ^13^C NMR spectroscopy and elemental analysis or HRMS (see Experimental and Supporting Information File 1). Some of them were characterized also by means of X-ray analysis (see below).

**Scheme 5:**
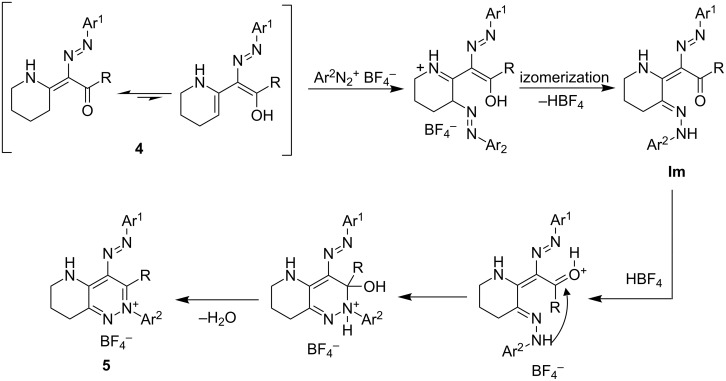
Possible mechanism of the formation of the pyridazinium salts **5**.

The mechanism of the azo coupling of primary and secondary enaminones was studied in [29]. The formation of compounds **4** can be assumed to proceed in an analogous way. A possible explanation of the formation of the pyridazinium salts **5** from the intermediates **4** is shown in Scheme 5.

Compound **4**, formed either in situ (methods A,B) or in a separate experiment (method C) can exist in equilibrium with its enol form. It reacts with another equivalent of the diazonium salt to form the product of double azo coupling **Im** (Scheme 5). We have observed the formation of similar compounds upon azo coupling of some enaminones [11,30–31]. Fluoroboric acid, liberated during the formation of **Im**, is not captured by the base (as no equivalent of sodium acetate is used in the 2nd step) and could catalyze an intramolecular attack of hydrazone NH nitrogen to the carbonyl group. After a subsequent loss of water, the molecule of pyridazinium salt **5** is formed. This hypothesis is supported by the fact that the use of an excess of sodium acetate led to significantly lower yields (for comparison see Table 1, method A). However, the above-mentioned scheme is only a speculation (as the intermediate **Im** has never been isolated) based on the observations made on similar reactions and on the knowledge of structures of the starting compounds and the products.

**Scheme 6:**
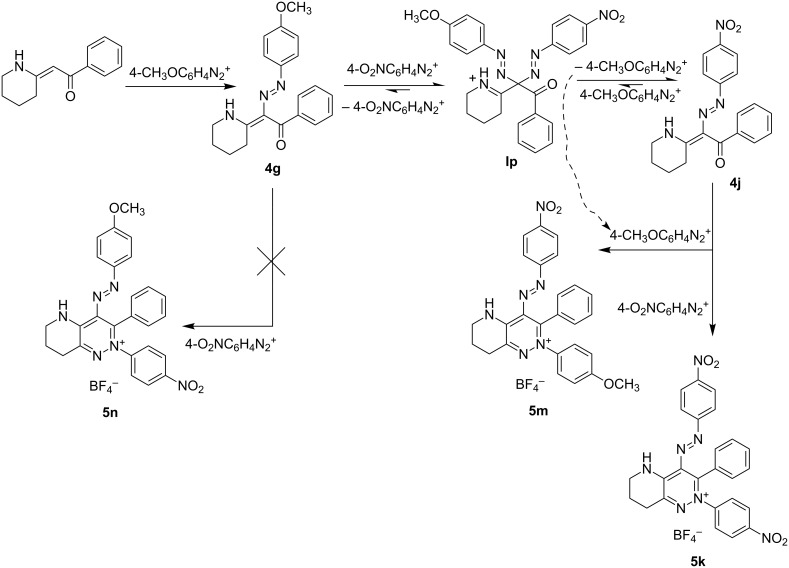
An attempt at synthesis of **5n** and possible explanation of the failure.

An attempt at preparation of 4-(4-methoxyphenyldiazenyl)-2-(4-nitrophenyl)-3-phenyl 5,6,7,8-tetrahydropyrido[3,2-*c*]pyridazin-2-ium tetrafluoroborate (**5n**) failed. A mixture of compounds **5k** + **5m** (approx. 1:2) was obtained instead. Crystallization of the mixture gave a mixed crystal (**5k** + **5m**, for discussion of its structure see below). A possible explanation for this lies in an exchange of the diazonium ions (Scheme 6).

The mechanism of the exchange is described in [32]. Its application to the reaction of **4g** with 4-nitrobenzenediazonium ion (Scheme 6) can explain the formation of the mixture of pyridazinium salts **5k** + **5m**: **4g** can react with another molecule of the diazonium salt either to give the bishydrazone intermediate **Im** and subsequently the pyridazinium salt **5n** (in the sense of Scheme 5) or it can undergo an *ipso* attack to form the intermediate **Ip**, consisting of 4-methoxy and 4-nitrophenyldiazenyl groups. The *ipso* attack is faster but reversible, whereas the formation of **Im** followed by the cyclization to the pyridazinium ion **5** is slower but irreversible. The less electrophilic benzenediazonium ion (4-methoxy) is preferentially eliminated from **Ip** to give **4j** (the product of the exchange of the diazenyl groups). **4j** can further react either with another equivalent of 4-nitrobenzenediazonium (to give **5k**) or with 4-methoxybenzenediazonium (formed by cleavage from the **Ip** intermediate) to give **5m** (both in the sense of Scheme 5). Compound **5n** is not formed as it requires the cleavage of the more electrophilic benzenediazonium ion (4-nitro) from **Ip** (disfavored).

The above-mentioned appears to be a drawback of the methodology: the more reactive diazonium salt should be used before the less reactive one otherwise a mixture of products can arise.

Similar results were obtained on reaction of **4b** with an equivalent of 4-bromobenzenediazonium tetrafluoroborate: a mixture of products was obtained. Attempts to separate the components of such mixtures failed, probably due to the very similar properties of the pyridazinium salts formed.

It should be emphasized that the above-mentioned suggestions on the mechanism are only suppositions based on the structures of the isolated compounds and reactants as well as the results obtained in similar reactions.

**Figure 1:**
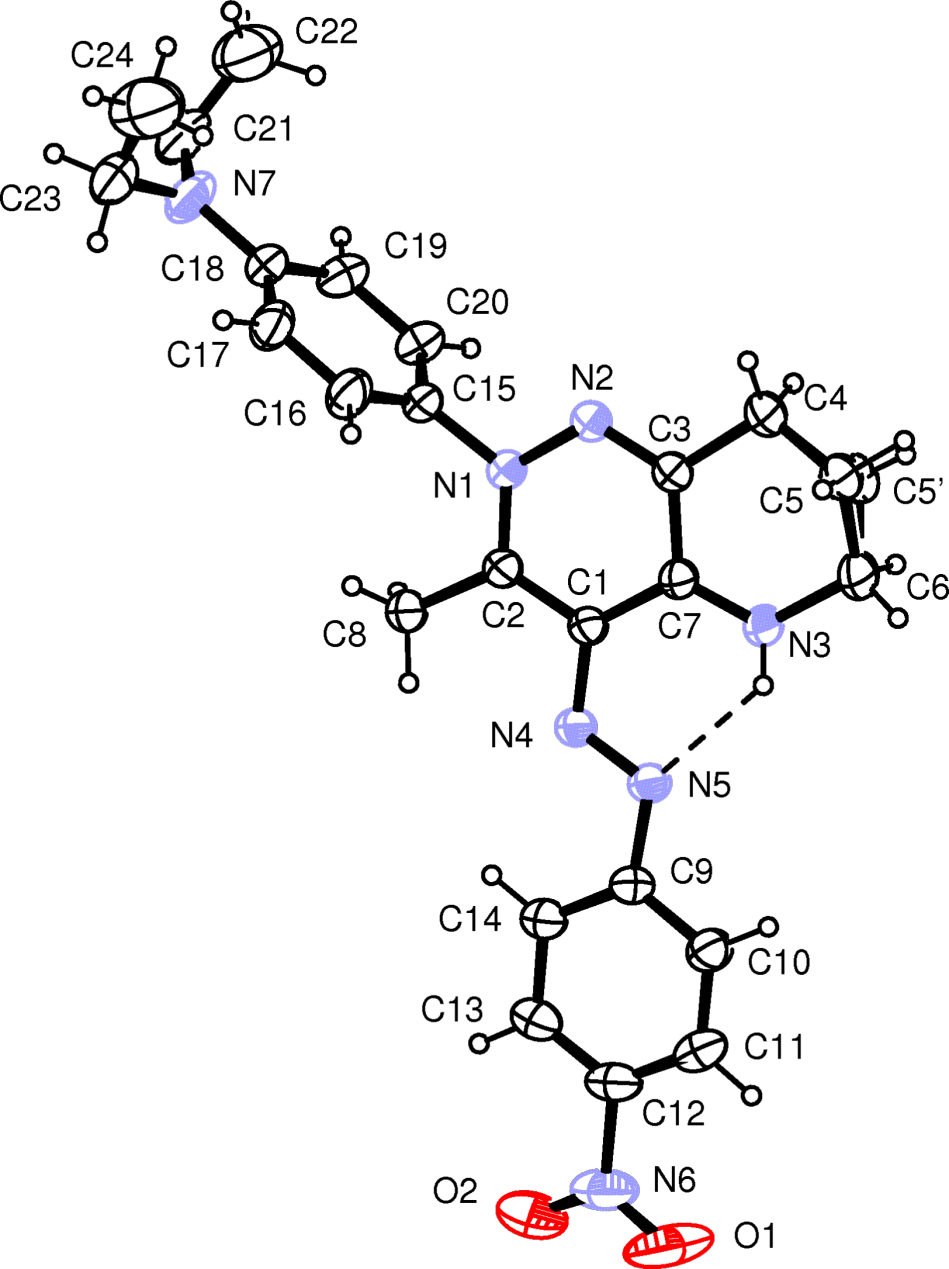
ORTEP view of the cation of compound **5f** showing the thermal ellipsoids at 30% probability level. Both the disordered C5H_2_ and C5’H_2_ moieties are displayed.

**Figure 2:**
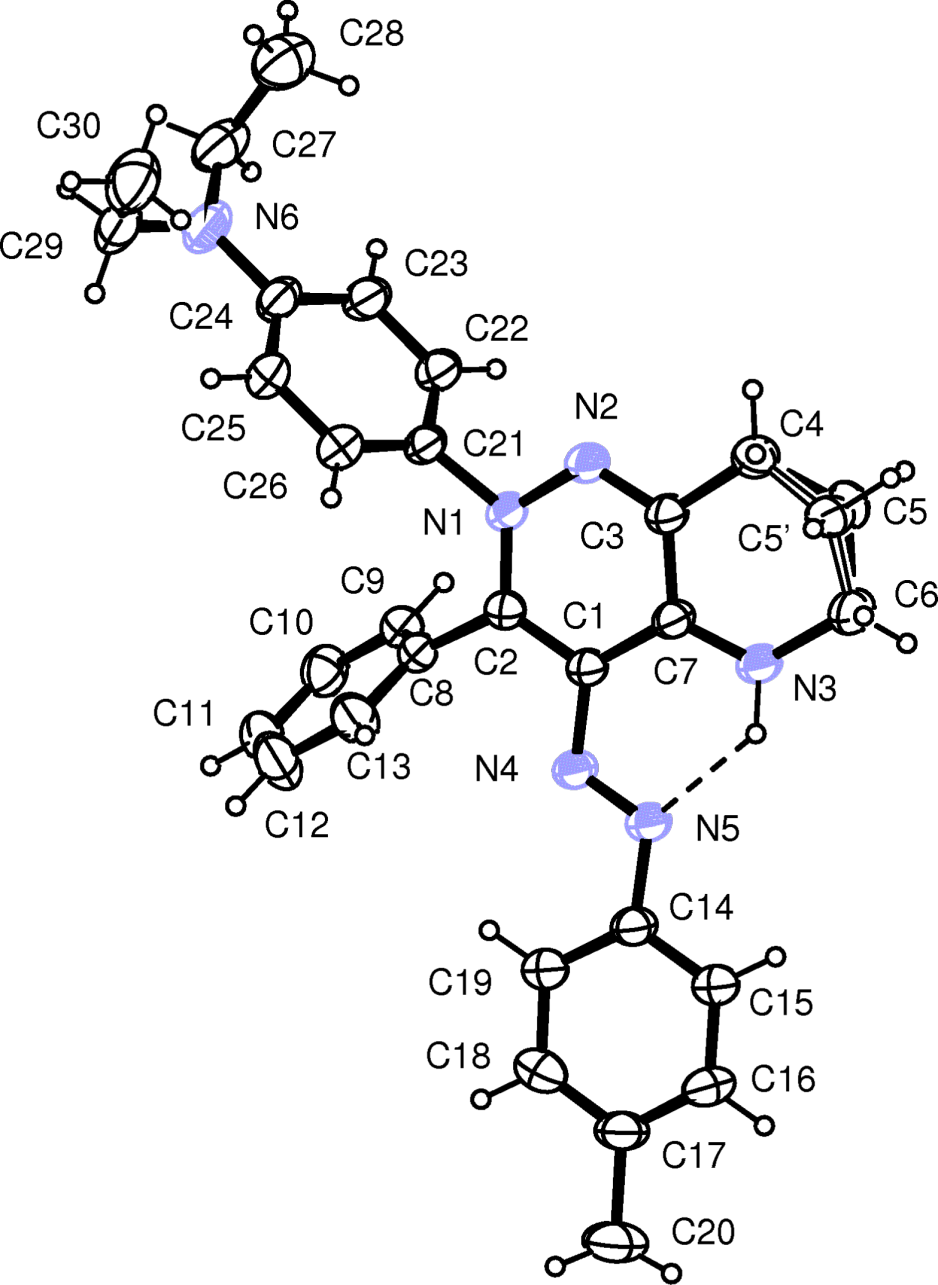
ORTEP view of the cation of compound **5l** showing the thermal ellipsoids at 30% probability level. Both the disordered C5H_2_ and C5’H_2_ moieties are displayed.

The structures of the compounds **5a**,**b**,**d**,**f**,**g**,**j**,**l**,**m** were also solved by using X-ray diffractometry. ORTEP [33] views of the compounds **5f** and **5l** are shown in Figure 1 and Figure 2, the others are given in Supporting Information File 1. Almost all the structures display some disorder. Structures **5d, 5f, 5j** and **5l** show disorder in the C3–C7,N3 six-membered rings and the C5H_2_ moieties were refined over two sites. All the BF_4_^−^ anions exhibit high temperature factors; in **5d** and **5l** the fluorine atoms were refined over two split sites. In **5m** the asymmetric unit is built up by two cations, which differ only in the conformation of the O–CH_3_ moiety, and two BF_4_^−^ anions. Also in **5k + 5m** the asymmetric unit is built up by two cations, but while the structure of the cation A did not present any problems during the refinement, the cation B revealed a structure with two superimposed substituents, –OCH_3_ and –NO_2_, in *para* position at C20b–C25b phenyl ring. The two different substituents were refined independently with occupancies of 0.5 each.

The crystal data are given in Supporting Information File 1 (Table S1). A selection of bond distances and angles and the intramolecular hydrogen bond parameters are also reported in Supporting Information File 1 (Table S2 and Table S3).

## Conclusion

We have developed an improved simple methodology for the construction of bicyclic pyridazinium tetrafluoroborates. The heterogeneous variant (method A) suffers from low yields, probably due to a partial decomposition of the diazonium salt during the reaction. An implementation of homogeneous reaction conditions using a methanolic solution of the corresponding diazonium tetrafluoroborates led to a substantial increase of both yields and the scope of the existing procedure. Two variations of the homogeneous procedure were tested:

1. A two-step procedure (method C) with the isolation and characterization of the intermediates **4** followed by their reaction with another equivalent of the diazonium salt.
2. A one-pot procedure (method B) with the addition of another equivalent of the diazonium salt into the reaction mixture directly after the consumption of the first equivalent without the isolation of the intermediate **4**.

The single-stage protocol (method B) is superior to the other ones as it gives comparable or better yields without the need to isolate the intermediate **4**.

Pyridazinium salts **5** arise from azo-coupled enaminones **4** probably via the products of double azo coupling (**Im**) through an intramolecular nucleophilic addition followed by the loss of water. The statement is, however, based only on the structures of the reactants and products and on the comparison with similar reactions.

A drawback of the method is the fact that azo coupling is a reversible reaction. The more reactive diazonium salt can replace the less reactive one to give a mixture of products. The ideal combination is to use the more reactive diazonium salt followed by the less reactive one, as the diazonium exchange is suppressed here.

## Experimental

All the solvents and reagents were purchased from commercial suppliers and were used without further purification. Diazonium tetrafluoroborates were prepared according to an ordinary procedure (diazotization using cold NaNO_2_/HCl with subsequent precipitation, from the filtered solution of the diazonium chloride, by aqueous sodium tetrafluoroborate). Precipitated diazonium tetrafluoroborates were dried in vacuo in a desiccator and stored in a refrigerator.

Compound **6** was prepared from δ-valerolactam and dimethyl sulfate according to the procedure described in [34]. Compounds **3a**,**b** and **7** were prepared according to the procedures published in [12].

NMR spectra were measured using a Bruker AVANCE III spectrometer operating at 400.13 (^1^H) and 100.62 MHz (^13^C). Some spectra were also measured using a Bruker Avance 500 spectrometer operating at 500.13 (^1^H) and 125.77 MHz (^13^C). All the pulse sequences were taken from the Bruker software library. Proton spectra measured in CDCl_3_ were calibrated based on internal TMS (δ = 0.00) and (in DMSO-*d*_6_) based on the middle signal of the solvent multiplet (δ = 2.55). Carbon NMR spectra were calibrated on the middle signal of the solvent multiplet (δ = 77.23 (CDCl_3_) and 39.60 (DMSO-*d*_6_)).

Elemental analyses were performed on a Flash 2000 CHNS Elemental Analyzer. Melting points were measured on a Kofler hot-stage microscope Boetius PHMK 80/2644 and were not corrected. MALDI HRMS were measured using a MALDI LTQ Orbitrap XL (Thermo Fisher Scientific) with DCTB as the matrix dissolved in acetonitrile. The crystal data of all the compounds were collected at room temperature using a Nonius Kappa CCD diffractometer with graphite monochromated Mo Kα radiation. The data sets were integrated with the Denzo-SMN package [35] and corrected for Lorentz, polarization and absorption effects (SORTAV) [36]. The structures were solved by direct methods using the SIR97 [37] system of programs and refined anisotropically by using full-matrix least-squares for all non-hydrogen atoms and hydrogen atoms included on their calculated positions, riding on their carrier atoms; except the N–H hydrogens forming intramolecular hydrogen bonds, which were refined isotropically. In the structure **5m** an ill-defined region of residual electron density was found. For this reason the program SQUEEZE was used to cancel out the effects of the undetermined disordered solvent. SQUEEZE is a part of the PLATON system of programs. All calculations were performed by using SHELXL-97 [38], PARST [39] and PLATON [40] implemented in WINGX [41] system of programs. Crystallographic data (excluding structure factors) have been deposited at the Cambridge Crystallographic Data Centre and allocated the deposition numbers CCDC 928078-928086. These data can be obtained free of charge via http://www.ccdc.cam.ac.uk/conts/retrieving.html or on application to CCDC, Union Road, Cambridge, CB2 1EZ, UK [fax: (+44)1223-336033, e-mail: deposit@ccdc.cam.ac.uk].

### Typical procedure for the synthesis of compounds **4**

Enaminone **3a** (5 mmol) and AcONa (5 mmol) were dissolved in cold (5 °C) methanol. A saturated methanolic solution of 4-methylbenzenediazonium tetrafluoroborate (5 mmol) was added dropwise over 30 minutes. The mixture was then stirred at ambient temperature for 24 h. The solvent was evaporated in vacuo, and the crude product was dissolved in dichloromethane and subjected to flash chromatography on silica gel. Subsequent crystallization from *n*-hexane/ethanol afforded the pure products.

**1-[2-(4-Methylphenyl)diazen-1-yl]-1-(piperidin-2-ylidene)propan-2-one** (**4a, R = Me, Ar^1^ = 4-MePh**) yield 89%, yellow crystals, mp 92–93 °C; ^1^H NMR (400 MHz, CDCl_3_) δ 15.18 (br s, 1H), 7.42–7.41 (br m, 2H), 7.16–7.14 (br m, 2H), 3.46 (br s, 2H), 3.08–3.06 (m, 2H), 2.50 (s, 3H), 2.33 (s, 3H), 1.71–1.73 (m, 4H) ppm; ^13^C NMR (101 MHz, CDCl_3_) 197.7, 162.5, 148.5, 135.9, 129.5, 128.1, 119.2, 42.3, 28.2, 28.0, 21.0, 20.7, 18.6 ppm; Anal. calcd for C_15_H_19_N_3_O: C, 70.01; H, 7.44; N, 16.33; found: C, 69.87; H,7.40; N, 16.53.

### Typical procedures for the synthesis of pyridazinium salts **5**

**Method A:** Solid 4-nitrobenzenediazonium tetrafluoroborate (5 mmol) was added in one portion into a cold (5 °C) mixture of enaminone **3a** (5 mmol) and sodium acetate (5 mmol) in dichloromethane (50 mL). After the diazonium salt had been consumed (negative test on chromotropic acid), an equivalent of 4-diethylaminobenzenediazonium tetrafluoroborate (5 mmol) was immediately added. The reaction mixture was then stirred at ambient temperature for 4 days. Sodium acetate was removed by suction, and the filtrate was evaporated to dryness in vacuo. The residue was dissolved in methanol/chloroform (1:10) and subjected to flash chromatography on silica gel. Subsequent crystallization from methanol afforded the desired product.

**Method B:** The procedure is analogous to method A, only methanol was used as the solvent and the diazonium salts were added dropwise (as saturated methanolic solutions) into the reaction mixture. The second equivalent of the diazonium salt was added after the consumption of the first one (negative test on chromotropic acid). The solvent was then evaporated in vacuo and the crude was purified by flash chromatography and crystallization.

**Method C:** Saturated methanolic solution of 4-diethylaminobenzenediazonium tetrafluoroborate (5 mmol) was added dropwise into the cold (5 °C) methanolic solution (5 mmol, 20 mL) of azo compound **4e** (prepared according to the above-mentioned procedure) over 30 minutes. The reaction mixture was stirred for 4 days at room temperature. The solvent was then evaporated in vacuo. The residue was dissolved in methanol/chloroform (1:10) and subjected to flash chromatography on silica gel. Subsequent crystallization from methanol afforded the desired product.

**2-(4-(Diethylamino)phenyl)-4-(4-nitrophenyldiazenyl)-3-methyl-5,6,7,8-tetrahydropyrido[3,2-*c*]pyridazin-2-ium tetrafluoroborate** (**5f**) Yields: A: 16%, B: 85%, C : 85%, red crystals, mp 150–152 °C; ^1^H NMR (400 MHz, DMSO-*d*_6_) δ 11.39 (br s, 1H), 8.48–8.45 (m, 2H), 8.36–8.34 (m, 2H), 7.43–7.41 (m, 2H), 6.85–6.83 (m, 2H), 3.43 (q, *J* = 7.0 Hz, 4H), 3.08–3.05 (m, 2H), 2.86 (s, 3H), 2.17–2.11 (m, 2H), 1.15 (t, *J* = 7.0 Hz, 6H); ^13^C NMR (101 MHz, DMSO-*d*_6_): 158.6, 155.0, 151.4, 148.8, 148.3, 137.1, 129.6, 126.8, 125.1, 124.0, 111.0, 43.9, 42.2, 27.6, 18.2, 17.0, 12.3; Anal. calcd for C_24_H_28_BF_4_N_7_O_2_: C, 54.05; H, 5.29; N, 18.38; found: C, 54.01; H, 5.45; N, 18.10.

## Supporting Information

File 1 Experimental procedures, characterization data and X-ray parameters.
