# Supplementary material for: A simple, enaminone-based approach to some bicyclic pyridazinium tetrafluoroborates
Source: Beilstein J Org Chem. 2013 Jul 23;9:1463–71. doi: 10.3762/bjoc.9.166 (PMC3740584; doi:10.3762/bjoc.9.166)
Supplement: File 1 — Experimental procedures, characterization data and X-ray parameters. [file Beilstein_J_Org_Chem-09-1463-s001.pdf]

Supporting Information

for

A simple, enaminone-based approach to some bicyclic pyridazinium tetrafluoroborates

František Josefík[1], Markéta Svobodová[1], Valerio Bertolasi[2] and Petr Šimůnek*[1]

Address: [1] Institute of Organic Chemistry and Technology, Faculty of Chemical Technology, University of Pardubice, Studentská 573, CZ 532 10, Pardubice, Czech Republic and [2] Dipartimento di Scienze Chimiche e Farmaceutiche, Centro di Strutturistica Diffratometrica, Università di Ferrara, Via L. Borsari 46, 441 00, Ferrara, Italy

Email: Petr Šimůnek - petr.simunek@upce.cz

*Corresponding author

Experimental procedures, characterization data and X-ray parameters

General

All the solvents and reagents were purchased from commercial suppliers and were used without further purification. Diazonium tetrafluoroborates were prepared according to a typical procedure (diazotization using cold $NaNO_2$/HCl with subsequent precipitation from the filtered solution of the diazonium chloride by aqueous sodium tetrafluoroborate). Precipitated diazonium tetrafluoroborates were dried in vacuo in a desiccator and stored in a refrigerator.

Compound **6** was prepared from δ-valerolactam and dimethyl sulfate according to the procedure described in [1]. Compounds **3a**,**b** and **7** were prepared according to the procedures published in [2].

NMR spectra were measured using Bruker AVANCE III spectrometer operating at 400.13 (^{1}H) and 100.62 (^{13}C). Some spectra were also measured using Bruker Avance 500 operating at 500.13 MHz (^{1}H) and 125.77 MHz (^{13}C). All the pulse sequences were taken from Bruker software library. Proton spectra measured in $CDCl_3$ were calibrated on internal TMS (δ = 0.00) and in DMSO-d_6 on the middle signal of the solvent multiplet (δ = 2.55). Carbon NMR spectra were calibrated on the middle signal of the solvent multiplet (δ = 77.23 ($CDCl_3$) and 39.60 (DMSO-d_6).

Elemental analyses were performed on Flash 2000 CHNS Elemental Analyzer. Melting points were measured on a Kofler hot-stage microscope Boetius PHMK 80/2644 and were not corrected.

MALDI HRMS were measured using a MALDI LTQ Orbitrap XL (Thermo Fisher Scientific) with DCTB as the matrix dissolved in acetonitrile.

The crystal data of all compounds were collected at room temperature using a Nonius Kappa CCD diffractometer with graphite monochromated Mo Kα radiation. The data sets were integrated with the Denzo-SMN package [3] and corrected for Lorentz,

polarization and absorption effects (SORTAV) [4]. The structures were solved by direct methods using the SIR97 [5] system of programs and refined using full-matrix least-squares with all non-hydrogen atoms anisotropically, and hydrogens were included on calculated positions, riding on their carrier atoms, except the N–H hydrogens forming intramolecular hydrogen bonds, which were refined isotropically. In the structure **5m** an ill-defined region of residual electron density was found. For this reason the program SQUEEZE was used to cancel out the effects of the undetermined disordered solvent. SQUEEZE is part of the PLATON system of programs. All calculations were performed using SHELXL-97 [6] , PARST [7] and PLATON [8] implemented in WINGX [9] system of programs. Crystallographic data (excluding structure factors) have been deposited at the Cambridge Crystallographic Data Centre and allocated the deposition numbers CCDC 928078-928086. These data can be obtained free of charge via www.ccdc.cam.ac.uk/conts/retrieving.html or on application to CCDC, Union Road, Cambridge, CB2 1EZ, UK [fax: (+44)1223-336033, e-mail: deposit@ccdc.cam.ac.uk].

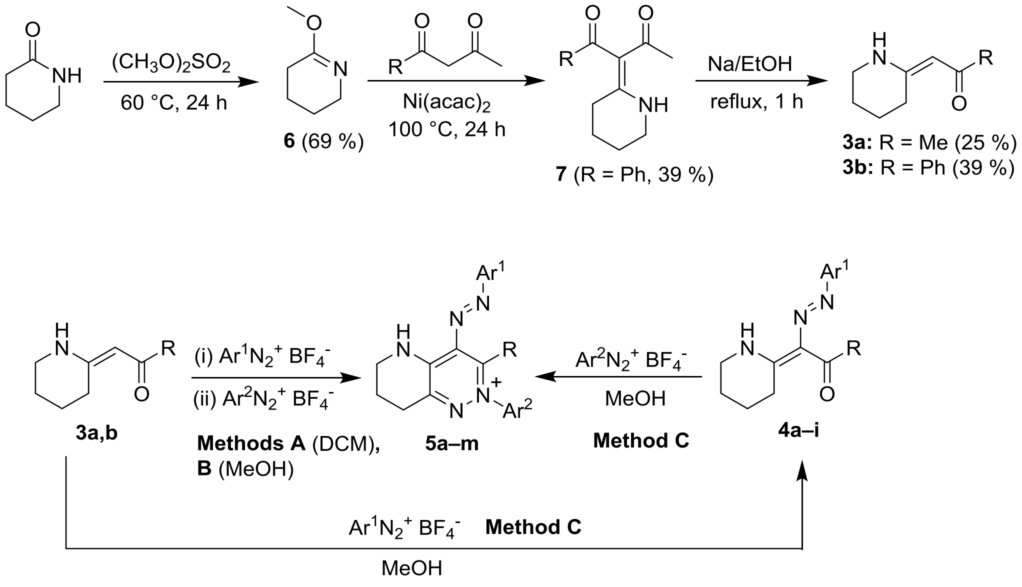

Scheme S1: Synthesis of the starting components and pyridazinium salts.

Typical procedure for the synthesis of compounds 4

Enaminone **3a** (5 mmol) and AcONa (5 mmol) were dissolved in cold (5 °C) methanol. A saturated methanolic solution of 4-methylbenzenediazonium tetrafluoroborate (5 mmol) was added dropwise over 30 minutes. The mixture was then stirred at ambient temperature for 24 h. Solvent was evaporated in vacuo, and the crude product was dissolved in dichloromethane and subjected to a flash chromatography on silica gel. Following crystallization from *n*-hexane/ethanol afforded pure products.

1-[2-(4-Methoxyphenyl)diazen-1-yl]-1-(piperidin-2-ylidene)propan-2-one (**4b, R = Me, Ar^1 = 4-OMePh**): yield 84%, yellow crystals, mp 97–98 °C; ^{1}H NMR (400 MHz, $CDCl_3$) δ 14.93 (s, 1H); 7.53–7.50 (m, 2H); 6.92–6.90 (m, 2H); 3.81 (s, 3H); 3.50–3.47 (br m, 2H); 3.13 (br t, *J* = 5.7, 2H); 2.51 (s, 3H); 1.86–1.70 (m, 4H); ^{13}C NMR (101 MHz, $CDCl_3$) δ 197.9; 161.6; 158.5; 145.6; 127.6; 121.0; 114.2; 55.5; 41.9; 28.1; 28.2; 20.8; 18.7; Anal. Calcd. for $C_{15}H_{19}N_3O_2$: C, 65.91; H, 7.01; N, 15.37; found: C, 65.89; H, 6.96; N, 15.57.

1-[2-(4-Bromophenyl)diazen-1-yl]-1-(piperidin-2-ylidene)propan-2-one (**4c, R = Me, Ar^1 = 4-BrPh**): yield 78%, orange crystals, mp 105–106 °C; ^{1}H NMR (400 MHz, $CDCl_3$) δ 15.19 (br s, 1H); 7.47–7.45 (m, 2H); 7.40–7.38 (m, 2H); 3.59–3.47 (m, 2H), 3.16–3.03 (m, 2H); 2.49 (s, 3H); 1.86–1.69 (m, 4H); ^{13}C NMR (101 MHz, $CDCl_3$) δ 197.7; 163.2; 150.0; 132.0; 128.6; 121.1; 119.3; 42.5; 28.3; 20.8; 18.7; Anal. Calcd. for $C_{14}H_{16}N_3O$: C, 52.19; H, 5.01; N, 13.04; found: C, 52.30; H, 4.99; N, 13.23.

1-[2-(4-(Diethylamino)phenyl)diazen-1-yl]-1-(piperidin-2-ylidene)propan-2-one (**4d, R = Me, Ar^1 = 4-Et$_2$NHPh**): yield 76%, red crystals, mp 78–80°C; ^{1}H NMR (400 MHz, $CDCl_3$) δ 14.70 (br s, 1H); 7.51–7.47 (m, 2H); 6.70–6.64 (m, 2H); 3.47–3.45 (m, 2H), 3.38 (q, *J* = 6.9, 4H); 3.17–3.13 (m, 2H); 2.51 (s, 3H); 1.81–1.72 (m, 4H); 1.18 (t, *J* = 6.9, 6H); ^{13}C NMR (101 MHz, $CDCl_3$) δ 197.7; 161.3; 147.2; 142.4; 127.1; 121.7; 111.9;

44.6; 41.6; 28.4; 28.1; 21.1; 19.0; 12.7; HRMS: 313.20311 ($[M - H]^+$, $C_{18}H_{25}N_4O^+$; calc. 313.20229).

1-[2-(4-Nitrophenyl)diazen-1-yl]-1-(piperidin-2-ylidene)propan-2-one (**4e, R = Me, Ar^1 = 4-NO_2Ph**): yield 88%, red crystals, mp 126–127°C; ^{1}H NMR (400 MHz, $CDCl_3$) δ 15.58 (s, 1H); 8.23–8.20 (m, 2H); 7.55–7.53 (m, 2H); 3.74–3.62 (m, 2H); 3.11–3.08 (m, 2H); 2.52 (s, 3H); 1.89–1.75 (m, 4H); ^{13}C NMR (101 MHz, $CDCl_3$) δ 197.9; 165.4; 154.9; 144.5; 131.1; 125.2; 118.8; 43.7; 28.8; 28.1; 20.7; 18.6; Anal. Calcd. for $C_{14}H_{16}N_4O_3$: C, 58.32; H, 5.59; N,19.43; found: C, 58.45; H, 5.57; N, 19.68.

2-[2-(4-Methylphenyl)diazen-1-yl]-1-phenyl-2-(piperidin-2-ylidene)ethan-1-one (**4f, R = Ph, Ar^1 = 4-MePh**): yield 95%, yellow oil. ^{1}H NMR (400 MHz, $CDCl_3$) δ 15.15 (br s, 1H); 7.75–7.72 (m, 2H); 7.40–7.31 (m, 3H); 7.19–7.16 (m, 2H); 7.05–7.01 (m, 2H); 3.44–3.41 (br m, 2H); 3.07–3.04 (br m, 2H); 2.24 (s, 3H); 1.67–1.64 (m, 4H); ^{13}C NMR (101 MHz, $CDCl_3$) δ 193.1; 163.9; 148.0; 141.7; 135.5; 129.9; 129.4; 128.0; 127.0; 119.2; 42.7; 27.9; 20.9; 20.7; 18.5. HRMS: 320.17524 ($[M + H]^+$, $C_{20}H_{22}N_3O^+$; calc. 320.17574).

2-[2-(4-Methoxyphenyl)diazen-1-yl]-1-phenyl-2-(piperidin-2-ylidene)ethan-1-one (**4g, R = Ph, Ar^1 = 4-OMePh**): yield 90%, yellow crystals, mp 92–93 °C; ^{1}H NMR (400 MHz, $CDCl_3$) δ 15.00 (br s, 1H); 7.71–7.69 (br m, 2H); 7.42–7.35 (m, 3H); 7.27–7.25 (m, 2H); 6.83–6.79 (m, 2H); 3.77 (s, 3H); 3.59–3.52 (m, 2H); 3.20–3.18 (m, 2H); 1.83–1.82 (m, 4H); ^{13}C NMR (101 MHz, $CDCl_3$) δ 193.7; 163.3; 158.5; 145.4; 142.2; 130.0; 129.9; 127.5; 127.2; 121.2; 114.2; 55.5; 42.4; 28.0; 21.1; 18.9; Anal. Calcd. for $C_{20}H_{21}N_3O_2$: C, 71.62; H, 6.31; N, 12.53; found: C, 71.59; H, 6.34; N, 12.31.

2-[2-(4-Bromophenyl)diazen-1-yl]-1-phenyl-2-(piperidin-2-ylidene)ethan-1-one (**4h, R = Ph, Ar^1 = 4-BrPh**): yield 58%, yellow crystals, mp 132–133 °C; ^{1}H NMR (400 MHz, $CDCl_3$) δ 15.23 (br s, 1H); 7.72–7.70 (m, 2H); 7.46–7.42 (m, 1H); 7.39–7.35 (m, 4H);

7.15–7.11 (m, 2H); 3.63–3,60 (m, 2H); 3.13–3.11 (m, 2H); 1.90–1.74 (m, 4H); ^{13}C NMR (101 MHz, $CDCl_3$) δ 193.5; 164.7; 149.4; 141.4; 132.0; 130.4; 130.0; 128.8; 127.3; 120.9; 118.9; 43.3; 28.2; 21.0; 18.8; Anal. Calcd. for: $C_{19}H_{18}BrN_3O$: C, 59.39; H, 4.72; N, 10.94; found: C, 59.12; H, 4.66; N 10.92.

2-[2-[4-(Diethylamino)phenyl]diazen-1-yl]-1-phenyl-2-(piperidin-2-ylidene)ethan-1-one (**4i, R = Ph, Ar^1 = 4-Et$_2$NPh**): Product is too unstable to be isolated and was used directly in the next reaction step. HRMS: 376.22591 ($[M]^+$, $C_{23}H_{28}N_4O^+$; calc. 376.22576).

2-[2-[4-Nitrophenyl]diazen-1-yl]-1-phenyl-2-(piperidin-2-ylidene)ethan-1-one (**4j, R = Ph, Ar^1 = 4-NO$_2$NPh**): yield 93%, red crystals, mp 125–126 °C; ^{1}H NMR (400 MHz, $CDCl_3$) δ 15.66 (s, 1H), 8.12–8.09 (m, 2H), 7.81–7.79 (m, 2H), 7.54–7.50 (m, 1H), 7.44–7.41 (m, 2H), 7.26–7.23 (m, 2H), 3.77 (br s, 2H), 3.01–2.98 (br m, 2H), 1.84–1.83 (br m, 4H); ^{13}C NMR (101 MHz, $CDCl_3$) δ 193.0, 166.2, 153.6, 143.9, 139.9, 132.0, 131.4, 130.2, 127.6, 125.3, 117.9, 44.9, 28.5, 21.0, 18.6; Anal. Calcd. for: $C_{19}H_{18}N_4O_3$: C, 65.13; H, 5.18; N, 15.99; found: C, 65.10; H, 5.10; N 16.08.

Typical procedures for the synthesis of pyridazinium salts 5

Method A: Solid 4-nitrobenzenediazonium tetrafluoroborate (5 mmol) was at once added into the cold (5 °C) mixture of enaminone **3a** (5 mmol) and sodium acetate (5 mmol) in dichloromethane (50 mL). After the diazonium salt was consumed (negative test on chromotropic acid), an equivalent of 4-diethylaminobenzenediazonium tetrafluoroborate (5 mmol) was at once added. The reaction mixture was then stirred at ambient temperature for 4 days. Sodium acetate was removed by suction and the filtrate was evaporated to dryness in vacuo. The residue was dissolved in

methanol/chloroform (1:10) and subjected to a flash chromatography on silica gel. Subsequent crystallization from methanol afforded the desired product.

Method B: The procedure is analogous to the method A, only methanol was used as the solvent and the diazonium salts were added dropwise as saturated methanolic solutions into the reaction mixture. The second equivalent of the diazonium salt was added after the consumption of the first one (negative test on chromotropic acid).

Method C: Saturated methanolic solution of 4-diethylaminobenzenediazonium tetrafluoroborate (5 mmol) was added dropwise into the cold (5 °C) methanolic solution of azo compound **4e** (5 mmol, 20 mL) during 30 minutes. The reaction mixture was stirred for 4 days at room temperature. The solvent was then evaporated in vacuo. The residue was dissolved in methanol/chloroform (1:10) and subjected to a flash chromatography on silica gel. Subsequent crystallization from methanol afforded the desired product.

2-(4-Methylphenyl)-4-(4-methylphenyldiazenyl)-3-methyl-5,6,7,8-tetrahydropyrido[3,2-*c*]pyridazin-2-ium tetrafluoroborate (5a):

Yields: A: 40%, B: 70%, C: 41%, orange crystals, mp >260 °C; ^{1}H NMR (400 MHz, DMSO-d_6) δ 11.37 (s, 1H), 8.13–8.11 (m, 2H), 7.62–7.60 (m, 2H), 7.55–7.49 (m, 4H), 3.79 (br t, J = 5.2 Hz, 2H), 3.09 (br t, J = 6.3, 2H), 2.82 (s, 3H), 2.49 (s, 3 H), 2.48 (s, 3H), 2.17–2.14 (m, 2H); ^{13}C NMR (101 MHz, DMSO-d_6) δ 157.1, 150.6, 150.1, 143.6, 140.5, 139.9, 137.9, 130.5, 130.2, 126.3, 126.1, 123.3, 42.0, 27.5, 21.2, 21.0, 18.1, 16.9; Anal. Calcd. for: $C_{22}H_{24}BF_4N_5$: C, 59.34; H, 5.43; N, 15.73; found: C, 59.14; H, 5.42; N, 15.52.

2-(4-Methoxyphenyl)-4-(4-methoxyphenyldiazenyl)-3-methyl-5,6,7,8-tetrahydropyrido[3,2-*c*]pyridazin-2-ium tetrafluoroborate (5b):

Yields: A: 39%, B: 92%, C: 81%, yellow crystals, mp 258–260 °C (dec.); ^{1}H NMR (500 MHz, DMSO-d_6) δ 11.30 (s, 1H), 8.23–8.20 (m, 2H), 7.67–7.64 (m, 2H), 7.27–7.22 (m, 4H), 3.95 (s, 3H), 3.92 (s, 3H), 3.79–3.76 (m, 2H), 3.40 (s, 3H), 3.08 (t, *J* = 6.4 Hz, 2H), 2.17–2.12 (m, 2H); ^{13}C NMR (125 MHz, DMSO-d_6) δ 163.3, 160.4, 156.9, 150.3, 146.3, 137.9, 135.2, 127.6, 126.5, 125.5, 115.0 (2 ×C), 56.0, 55.8, 42.0, 27.6, 18.3, 16.8; Anal. Calcd. for: $C_{22}H_{24}BF_4N_5O_2$: C, 55.36; H, 5.07; N, 14.67; found: C, 55.21; H, 5.06; N 14.39.

2-(4-Bromophenyl)-4-(4-bromophenyldiazenyl)-3-methyl-5,6,7,8-tetrahydropyrido[3,2-*c*]pyridazin-2-ium tetrafluoroborate (5c):

Yields: A: 47%, B: 60%, C: 61%), orange crystals, mp >260°C; ^{1}H NMR (400 MHz, DMSO-d_6) δ 11.42 (s, 1H), 8.19–8.15 (m, 2H), 7.99–7.96 (m, 2H), 7.93–7.90 (m, 2H), 7.73–7.70 (m, 2H), 3.79 (br t, *J* = 5.4 Hz, 2H), 3.10 (br t, *J* = 6.2 Hz, 2H), 2.84 (s, 3H), 2.17–2.13 (m, 2H); ^{13}C NMR (101 MHz, DMSO-d_6) δ 157.9, 151.2, 150.9, 141.3, 138.0, 133.2, 132.9, 128.6, 126.6, 126.4, 125.1, 124.1, 42.3, 27.6, 18.2, 16.9; HRMS: 485.99324 ($[M]^+$, $C_{20}H_{18}Br_2N_5^+$; calc. 485.99235).

2-(4-(Diethylamino)phenyl)-4-(4-(diethylamino)phenyldiazenyl)-3-methyl-5,6,7,8-tetrahydropyrido[3,2-*c*]pyridazin-2-ium tetrafluoroborate (5d):

Yields: A: 24%, B: 45%, C: 50%, red crystals, mp 153–155 °C; ^{1}H NMR (400 MHz, DMSO-d_6) δ 11.20 (br s, 1H), 8.05–8.03 (m, 2H), 7.43–7.41 (m, 2H), 6.91–6.89 (m, 2H), 6.86–6.84 (m, 2H), 3.79–3.69 (br m, 2H), 3.56 (q, *J* = 7.0 Hz, 4H), 3.47 (q, *J* = 7.0 Hz, 4H), 3.05–3.02 (m, 2H), 2.78 (s, 3H), 2.17–2.09 (m, 2H), 1.24–1.17 (m, 12H); ^{13}C NMR (101 MHz, DMSO-d_6) δ 154.6, 151.4, 148.7, 148.1, 142.2, 137.6, 130.0, 127.2, 126.8,

126.1 (br), 111.4, 110.9, 44.3, 43.9, 41.5, 27.4, 18.3, 16.6, 12.5, 12.2; HRMS: 472.31942 ($[M]^+$, $C_{28}H_{38}N_7^+$; calc. 472.31832).

2-(4-Nitrophenyl)-4-(4-nitrophenyldiazenyl)-3-methyl-5,6,7,8-tetrahydropyrido[3,2-*c*]pyridazin-2-ium tetrafluoroborate (5e):

Yields: A: 24%, B: 57%, C: 79%, brown crystals, mp >260 °C; ^{1}H NMR (400 MHz, DMSO-d_6) δ 11.59 (s, 1H), 8.63–8.59 (m, 2H); 8.56–8.53 (m, 2H), 8.46–8.44 (m, 2H), 8.09–8.05 (m, 2H), 3.87–3.82 (m, 2H), 3.13 (br t, *J* = 6.4 Hz, 2H), 2.91 (s, 3H), 2.20–2.17 (m, 2H); ^{13}C NMR (101 MHz, DMSO-d_6) δ 158.8, 155.1, 152.0, 149.1, 148.5, 146.4, 138.0, 128.3, 126.7, 125.7, 125.3, 124.2, 42.6, 27.6, 18.2, 17.0; HRMS: 420.14149 ($[M]^+$, $C_{20}H_{18}N_7O_4^+$; calc. 420.14093).

3-Phenyl-2-(4-methylphenyl)-4-(4-methylphenyldiazenyl)-5,6,7,8-tetrahydropyrido[3,2-*c*]pyridazin-2-ium tetrafluoroborate (5g):

Yields: A: 50%, B: 80%, C : 71%, orange crystals, mp 250–251 °C; ^{1}H NMR (400 MHz, DMSO-d_6) δ 11.36 (s, 1H), 7.67–7.65 (m, 2H), 7.52–7.50 (m, 2H), 7.47–7.41 (m, 5H), 7.38–7.36 (m, 2H), 7.26–7.24 (m, 2H), 3.88–3.85 (m, 2H), 3.20 (br t, *J* = 6.5 Hz, 2H), 2.39 (s, 3H), 2.31 (s, 3H), 2.26–2.21 (m, 2H); ^{13}C NMR (101 MHz, DMSO-d_6) δ 156.9, 151.3, 150.0, 143.4, 140.5, 139.4, 138.8, 130.5, 130.1, 130.0, 129.5, 127.6, 126.9, 126.6, 122.8, 42.2, 27.7, 21.1, 20.6, 18.1; Anal. Calcd. for: $C_{27}H_{26}BF_4N_5$: C, 63.92; H, 5.17; N, 13.80; found: C, 64.05; H, 5.09; N 13.54.

3-Phenyl-2-(4-methoxyphenyl)-4-(4-methoxyphenyldiazenyl)-5,6,7,8-tetrahydropyrido[3,2-*c*]pyridazin-2-ium tetrafluoroborate (5h):

Yields: A: 10%, B: 72%, C 74%, orange crystals, mp 255 °C (dec.); ^{1}H NMR (400 MHz, DMSO-d_6) δ 11.28 (s, 1H), 7.75–7.71 (m, 2H), 7.50–7.41 (m, 7H), 7.14–7.11 (m, 2H), 6.99–6.95 (m, 2H), 3.88 (s, 3H), 3.84 (br s, 2H), 3.77 (s, 3H), 3.22–3.18 (m, 2H), 2.23–2.20 (m, 2H); ^{13}C NMR (125 MHz, DMSO-d_6) δ 163.2, 159.6, 156.7, 151.0, 146.3,

138.9, 135.9, 130.5, 130.0, 129.8, 128.3, 127.8, 127.1, 125.2, 115.0, 114.1, 56.0, 55.6, 42.2, 27.8, 18.2; Anal. Calcd. for: $C_{27}H_{26}BF_4N_5O_2$: C, 60.13; H, 4.86; N, 12.99; found: C, 59.88; H, 4.84; N, 12.73.

3-Phenyl-2-(4-bromophenyl)-4-(4-bromophenyldiazenyl)-5,6,7,8-tetrahydropyrido[3,2-*c*]pyridazin-2-ium tetrafluoroborate (5i):

Yields: A: 40%, B: 53%, C: 55%, orange crystals, mp 225–227 °C; ^{1}H NMR (400 MHz, DMSO-d_6) δ 11.41 (s, 1H), 7.83–7.80 (m, 2H), 7.71–7.67 (m, 4H), 7.51–7.46 (m, 7H), 3.88–3.86 (m, 2H), 3.23–3.20 (m, 2H), 2.24–2.22 (m, 2H); ^{13}C NMR (101 MHz, DMSO-d_6) δ 170.4, 157.3, 151.8, 150.6, 141.9, 138.9, 132.7, 132.2, 130.6, 130.4, 129.0, 129.0, 127.9, 127.0, 126.5, 124.5, 123.0, 59.8, 42.4, 27.7, 20.8, 18.1, 14.1; Anal. Calcd. for: $C_{25}H_{20}BBr_2F_4N_5$: C, 47.13; H, 3.16; N, 10.99; found: C, 47.41; H, 3.34; N 10.71.

3-Phenyl-2-(4-(diethylamino)phenyl)-4-(4-(diethylamino)phenyldiazenyl)-5,6,7,8-tetrahydropyrido[3,2-*c*]pyridazin-2-ium tetrafluoroborate (5j):

Yield: A: 32%, C: 26%, red crystals, mp 250–252 °C; ^{1}H NMR (400 MHz, $CDCl_3$) δ 11.54 (s, 1H), 7.55–7.52 (m, 2H), 7.38–7.31 (m, 3H), 7.28–7.26 (m, 2H), 7.04–7.02 (m, 2H), 6.60–6.58 (m, 2H), 6.48–6.45 (m, 2H), 3.91–3.87 (m, 2H), 3.42 (q, J = 7.1 Hz, 4H), 3.30 (q, J = 7.1, 4H), 3.14 (br t, J = 6.3, 2H), 2.25–2.19 (m, 2H), 1.19 (t, J = 7.1, 6H), 1.11 (t, J = 7.0, 6H); ^{13}C NMR (101 MHz, $CDCl_3$) δ 154.5, 151.9, 149.6, 148.1, 143.1, 139.8, 131.0, 130.9, 130.2, 129.8, 128.2, 127.9, 127.3, 111.6, 111.0, 45.1, 44.6, 42.0, 28.2, 18.8, 12.8, 12.5; Anal. Calcd. for: $C_{33}H_{40}BF_4N_7$: C, 63.77; H, 6.49; N, 15.78; found: C, 63.51; H, 6.57; N, 15.51.

3-Phenyl-2-(4-nitrophenyl)-4-(4-nitrophenyldiazenyl)-5,6,7,8-tetrahydropyrido[3,2-*c*]pyridazin-2-ium tetrafluoroborate (5k):

Yield: A: 1%, B: 61%, C: 68%, red crystals, mp 250–252 °C; ^{1}H NMR (400 MHz, DMSO-d_6) δ 11.60 (br s, 1H), 8.46–8.44 (m, 2H), 8.36–8.34 (m, 2H), 7.97–7.95 (m, 2H), 7.86–

7.83 (m, 2H), 7.57–7.46 (m, 5H), 3.94–3.91 (br m, 2H), 3.26 (br t, *J* = 6.3, 2H), 2.29–2.23 (m, 2H); ^{13}C NMR (101 MHz, DMSO-d_6): 158.0, 154.7, 152.5, 148.9, 147.5, 147.1, 138.8, 130.9, 130.8, 128.61, 128.59, 128.1, 127.2, 125.2, 124.7, 123.7, 42.7, 27.7, 18.0; Anal. Calcd. for: $C_{25}H_{20}BF_4N_7O_4$: C, 52.75; H, 3.54; N, 17.22; found: C, 52.64; H, 3.43; N 16.95.

3-Phenyl-2-(4-(diethylamino)phenyl)-4-(4-methylphenyldiazenyl)-5,6,7,8-tetrahydropyrido[3,2-*c*]pyridazin-2-ium tetrafluoroborate (5l):

Yield: A: 15%, B: 93%, C: 73%, red crystals, mp >260 °C; ^{1}H NMR (400 MHz, DMSO-d_6) δ 11.27 (s, 1H), 7.65–7.63 (m, 2H), 7.51–7.44 (m, 5H), 7.37–7.35 (m, 2H), 7.26–7.23 (m, 2H), 6.61–6.59 (m, 2H), 3.85–3.83 (m, 2H), 3.33 (q, *J* = 7.1, 4H), 3.21–3.17 (m, 2H), 2.39 (s, 3H), 2.25–2.19 (m, 2H), 1.07 (t, *J* = 7.1, 6H); ^{13}C NMR (101 MHz, DMSO-d_6) δ 156.9, 151.2, 150.0, 147.5, 143.3, 138.3, 130.7, 130.4, 130.1, 129.9, 129.7, 127.6, 127.5, 127.0, 122.8, 110.4, 43.7, 42.1, 27.7, 21.1, 18.1, 12.2; Anal. Calcd. for: : $C_{30}H_{33}BF_4N_6$: C, 63.84; H, 5.89; N, 14.89; found: C, 64.00; H, 5.76; N, 14.66.

3-Phenyl-2-(4-methoxyphenyl)-4-(4-nitrophenyldiazenyl)-5,6,7,8-tetrahydropyrido[3,2-*c*]pyridazin-2-ium tetrafluoroborate (5m):

Yield: B: 76%, C: 85%, red crystals, mp 221–223 °C; ^{1}H NMR (400 MHz, DMSO-d_6) δ 11.50 (s, 1H), 8.42–8.40 (m, 2H), 7.94–7.92 (m, 2H), 7.57–7.47 (m, 7H), 7.00–6.98 (m, 2H), 3.93–3.89 (m, 2H), 3.77 (s, 3H), 3.25–3.22 (m, 2H), 2.30–2.23 (m, 2H); ^{13}C NMR (101 MHz, DMSO-d_6) δ 159.6, 158.4, 154.8, 152.3, 148.8, 138.4, 135.7, 130.5, 130.2, 129.3, 128.2, 127.8, 127.3, 125.1, 123.6, 114.1, 55.5, 42.5, 27.8, 18.1; Anal. Calcd. for: $C_{26}H_{23}BF_4N_6O_3$: C, 56.34; H, 4.18; N, 15.16; found: C, 56.10; H, 4.03; N 14.90.

Table S1: Crystallographic data.

Compound	**5a**	**5b**	**5d**	**5f**	**5g**
Formula	$[C_{22}H_{24}N_5]^+[BF_4]^-$	$[C_{22}H_{24}N_5O_2]^+[BF_4]^-$	$[C_{28}H_{38}N_7]^+[BF_4]^-$	$[C_{24}H_{28}N_7O_2]^+[BF_4]^-$	$[C_{27}H_{26}N_5]^+[BF_4]^-$
M	445.27	477.27	559.246	533.34	507.34
Space group	*P2₁/c*	*P-1*	*P-1*	*P2₁/c*	*Cc*
Crystal system	Monoclinic	Triclinic	Triclinic	Monoclinic	Monoclinic
a/Å	14.0030(5)	9.0497(3)	10.2178(2)	15.7940(3)	11.1843(3)
b/Å	9.7700(3)	9.7640(4)	11.0808(2)	9.7487(2)	24.1870(7)
c/Å	16.2127(6)	14.5783(7)	13.0354(4)	16.9654(3)	9.9083(4)
α/°	90.00	86.013(2)	86.2244(7)	90.00	90.00
β/°	93.790(2)	77.864(2)	86.1692(8)	95.888(2)	108.148(1)
γ/°	90.00	63.894(2)	89.5178(9)	90.00	90.00
$U/Å^3$	2213.2(1)	1130.50(8)	1469.38(6)	2598.40(9)	2547.0(1)
Z	4	2	2	4	4
T/K	295	295	295	295	295
D_c/g cm^{-3}	1.336	1.402	1.264	1.363	1.323
F(000)	928	496	592	1112	1056
μ(Mo-Kα)/cm^{-1}	1.05	1.14	0.95	1.09	1.00
Measured Reflections	7110	6619	10118	10443	10944
Unique Reflections	3878	3970	7015	5885	4926
R_{int}	0.0504	0.0346	0.0203	0.0215	0.0370
Obs. Refl.ns [I≥2σ(I)]	2302	2259	4975	4236	3928
$θ_{min}$- $θ_{max}$/°	3.65–25.00	2.60–25.00	4.34–28.00	4.42–27.50	3.37–27.00
hkl ranges	-16,16;-10,11;-19,19	-10,10;-11,11;-17,17	-10,13;-14,14;-17,16	-20,20;-12,11;-22,21	-14,14;-30,28;-12,12
$R(F^2)$ (Obs.Refl.ns)	0.0742	0.0658	0.0643	0.0776	0.0736
$wR(F^2)$ (All Refl.ns)	0.2390	0.2044	0.1947	0.2352	0.2218
No. Variables	362	315	407	360	340
Goodness of fit	1.031	1.024	1.058	1.039	1.043
$Δρ_{max}$; $Δρ_{min}$ /e $Å^{-3}$	0.68; -0.32	0.59; -0.28	0.31; -0.27	0.48; -0.24	0.75; -0.36
CCDC Deposition N.	928078	928079	928080	928081	928082

Table S1 contd. Crystallographic data.

Compound	**5j**	**5l**	**5m**	**5k + 5m**
Formula	$[C_{33}H_{40}N_7]^+[BF_4]^-$	$[C_{30}H_{33}N_6]^+[BF_4]^-$	$[C_{26}H_{23}N_6O_3]^+[BF_4]^-$	$[C_{26}H_{23}N_6O_3]^+$ $[C_{25.5}H_{21.5}N_{6.5}O_{3.5}]^+2[BF_4]^-$
M	621.53	564.43	554.32	1116.12
Space group	*P-1*	*P-1*	*C2/c*	*C2/c*
Crystal system	Triclinic	Triclinic	Monoclinic	Monoclinic
a/Å	10.4449(3)	10.1457(3)	47.1964(7)	47.8618(7)
b/Å	11.1382(3)	11.6268(4)	8.5355(1)	8.4340(1)
c/Å	14.6890(5)	14.4610(6)	27.7377(6)	27.5315(5)
α/°	106.923(1)	76.895(2)	90.00	90.00
β/°	100.260(1)	75.998(2)	109.5909(6)	109.6601(7)
γ/°	90.289(1)	78.700(2)	90.00	90.00
U/Å^3	1605.80(8)	1594.0(1)	10527.1(3)	10465.7(3)
Z	2	2	16	8
T/K	295	295	295	295
D_c/g cm^{-3}	1.285	1.176	1.399	1.417
F(000)	656	592	4576	4600
μ(Mo-Kα)/cm^{-1}	0.94	0.87	1.13	1.15
Measured Reflections	10332	9739	16416	16436
Unique Reflections	6263	5559	9149	10181
R_{int}	0.0458	0.0389	0.0307	0.0300
Obs. Refl.ns [I≥2σ(I)]	3107	2805	5716	5860
θ_{min}- θ_{max}/°	2.69–26.00	3.75–25.00	2.78–25.00	2.96–26.00
hkl ranges	-12,12;-13,11;-18,18	-12,12;-12,13;-17,17	-55,56;-8,10;-32,32	-57,58;-10,7;-33,33
R(F^2) (Obs.Refl.ns)	0.0644	0.0892	0.0761	0.0739
wR(F^2) (All Refl.ns)	0.1833	0.2836	0.2469	0.2309
No. Variables	424	414	732	753
Goodness of fit	1.016	0.967	1.030	1.021
$\Delta\rho_{max}$; $\Delta\rho_{min}$ /e Å^{-3}	0.20; -0.23	0.32; -0.38	0.71; -0.52	0.62; -0.37
CCDC Deposition N.	928083	928084	928085	928086

Table S2: Selected bond distances and angles (Å and degrees).

Distances	5a	5b	5d	5f	5g	5j	5l	5m	5m + 5k
N1-N2	1.359(4)	1.356(4)	1.353(2)	1.360(3)	1.369(4)	1.358(3)	1.351(4)	1.356(4)<br>1.352(4)	1.360(4)<br>1.363(3)
N1-C2	1.345(4)	1.344(4)	1.346(2)	1.345(3)	1.339(4)	1.351(3)	1.346(4)	1.350(4)<br>1.349(5)	1.355(4)<br>1.351(4)
N2-C3	1.302(5)	1.305(4)	1.302(2)	1.289(3)	1.297(5)	1.312(4)	1.303(4)	1.294(4)<br>1.305(5)	1.305(5)<br>1.302(4)
C1-C2	1.398(5)	1.391(4)	1.399(2)	1.389(3)	1.398(5)	1.396(4)	1.399(4)	1.391(4)<br>1.387(5)	1.392(5)<br>1.401(4)
C1-C7	1.415(5)	1.420(4)	1.415(3)	1.424(3)	1.431(4)	1.411(4)	1.403(5)	1.424(4)<br>1.425(5)	1.414(5)<br>1.423(4)
C3-C7	1.428(5)	1.434(4)	1.442(2)	1.441(3)	1.442(4)	1.433(4)	1.434(5)	1.450(5)<br>1.446(5)	1.441(5)<br>1.450(4)
N3-C6	1.460(5)	1.460(5)	1.460(3)	1.468(3)	1.479(5)	1.459(5)	1.455(5)	1.463(5)<br>1.468(5)	1.462(5)<br>1.466(4)
N3-C7	1.328(5)	1.320(4)	1.325(2)	1.308(3)	1.316(4)	1.330(4)	1.328(4)	1.316(4)<br>1.313(5)	1.320(5)<br>1.315(4)
C1-N4	1.406(4)	1.400(4)	1.404(2)	1.400(3)	1.403(4)	1.403(3)	1.404(4)	1.405(4)<br>1.398(5)	1.408(4)<br>1.400(4)
N4-N5	1.263(4)	1.264(4)	1.277(2)	1.260(3)	1.267(4)	1.280(3)	1.268(4)	1.264(4)<br>1.264(4)	1.266(4)<br>1.268(3)
N5-C9	1.425(4)	1.426(4)	1.405(2)	1.426(3)					
N5-C14					1.428(4)	1.408(4)	1.424(4)	1.423(4)<br>1.435(5)	1.425(4)<br>1.424(4)

Table S2 contd. Selected bond distances and angles (Å and degrees).

Angles	5a	5b	5d	5f	5g	5j	5l	5m	5m + 5k
C2-N1-N2	124.5(3)	124.0(3)	124.0(1)	123.9(2)	124.2(3)	123.9(2)	124.1(3)	123.8(3)<br>123.6(3)	123.8(2)<br>124.1(2)
N1-N2-C3	117.9(3)	118.4(3)	118.3(1)	118.5(2)	118.0(3)	118.0(2)	118.2(3)	118.4(3)<br>118.8(3)	118.3(3)<br>118.1(3)
C2-C1-C7	118.9(3)	118.9(3)	118.6(2)	119.6(2)	118.7(3)	118.6(3)	118.9(3)	118.7(3)<br>118.8(3)	119.4(3)<br>118.6(3)
C7-N3-C6	124.1(4)	124.2(3)	124.3(2)	124.1(2)	123.2(3)	123.9(3)	125.0(3)	123.7(3)<br>124.7(4)	125.1(4)<br>124.1(3)
C1-N4-N5	115.3(3)	116.2(3)	114.8(2)	116.1(2)	115.4(3)	115.0(2)	114.7(3)	115.5(3)<br>116.8(3)	116.3(3)<br>115.1(2)
N4-N5-C9	114.4(3)	113.4(3)	114.3(2)	113.0(2)					
N4-N5-C14					113.3(3)	113.3(2)	114.4(3)	114.3(3)<br>112.3(3)	112.1(3)<br>113.9(3)

Table S3: Hydrogen Bond parameters (Å and degrees).

	N3–H3	N3···N5	H3···N5	N3–H3···N5
**5a**	0.85(5)	2.666(5)	2.04(5)	130(4)
**5b**	0.81(4)	2.696(4)	2.09(3)	132(4)
**5d**	0.86(2)	2.671(2)	2.01(2)	133(2)
**5f**	0.80(3)	2.683(5)	2.08(3)	131(3)
**5g**	1.01(5)	2.689(4)	1.96(4)	126(3)
**5j**	0.88(4)	2.659(4)	1.99(4)	132(4)
**5l**	0.80(4)	2.684(4)	2.09(4)	131(4)
**5m**	0.86(4)<br>0.90(5)	2.689(4)<br>2.729(5)	2.04(5)<br>2.13(4)	132(4)<br>123(4)
**5m+5k**	0.83(5)<br>0.90(4)	2.741(5)<br>2.690(4)	2.14(4)<br>1.97(5)	129(4)<br>135(4)

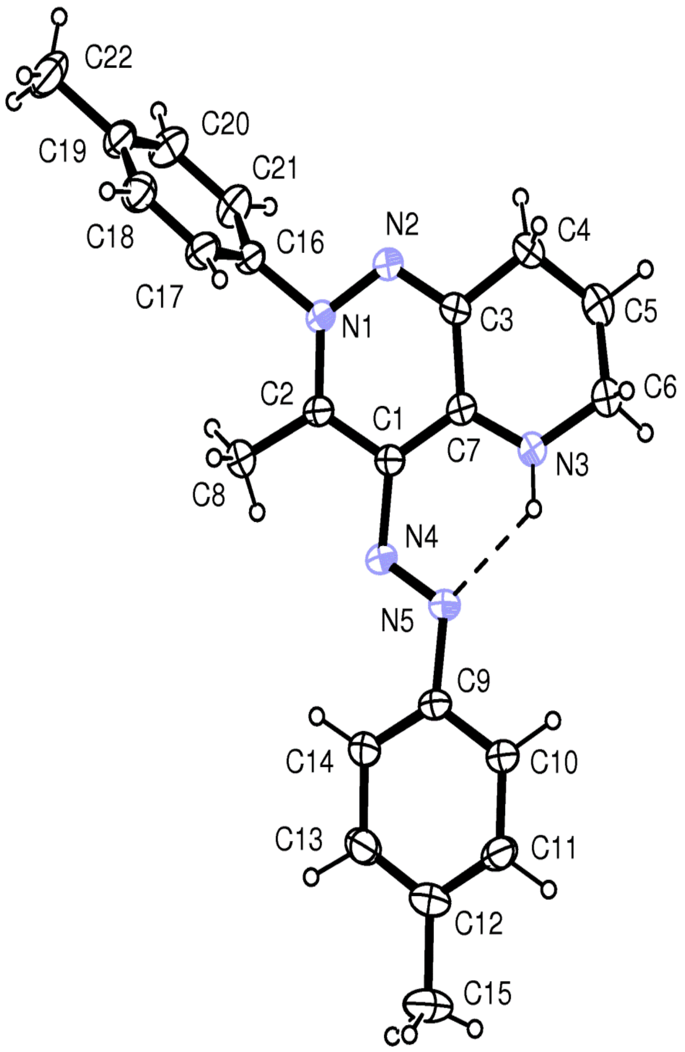

Figure S1: ORTEP view of the cation of compound **5a** showing the thermal ellipsoids at 30% probability level.

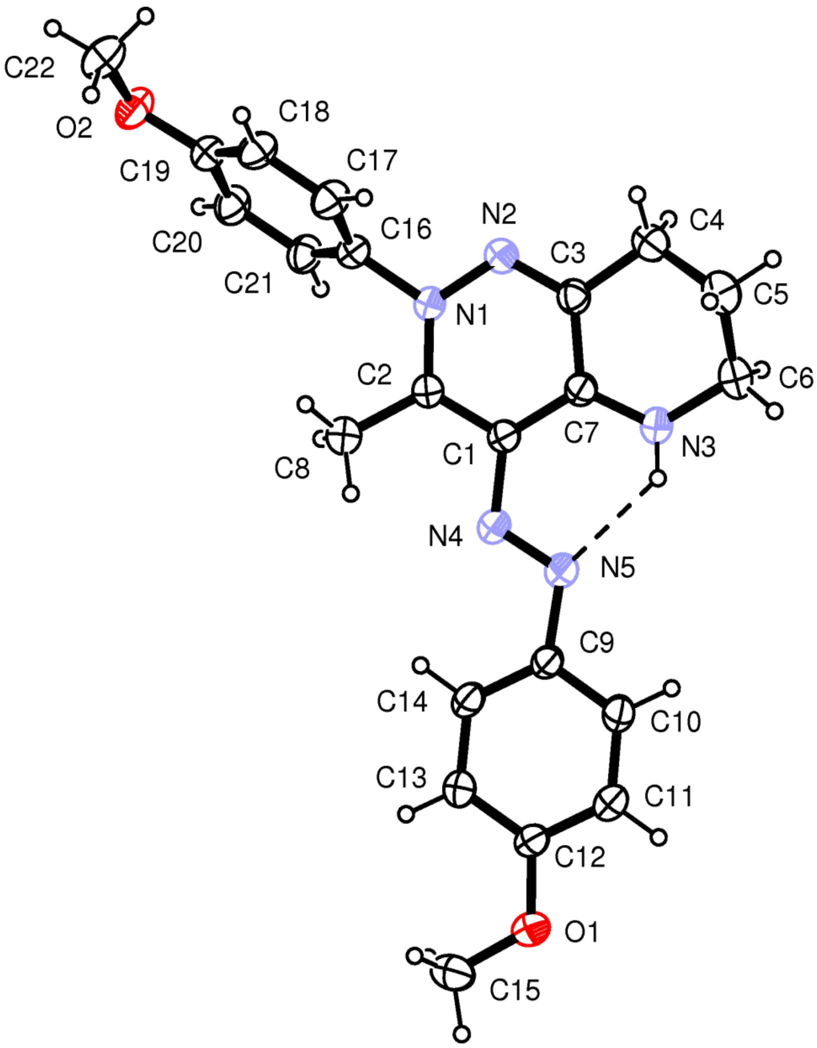

Figure S2: ORTEP view of the cation of compound **5b** showing the thermal ellipsoids at 30% probability level.

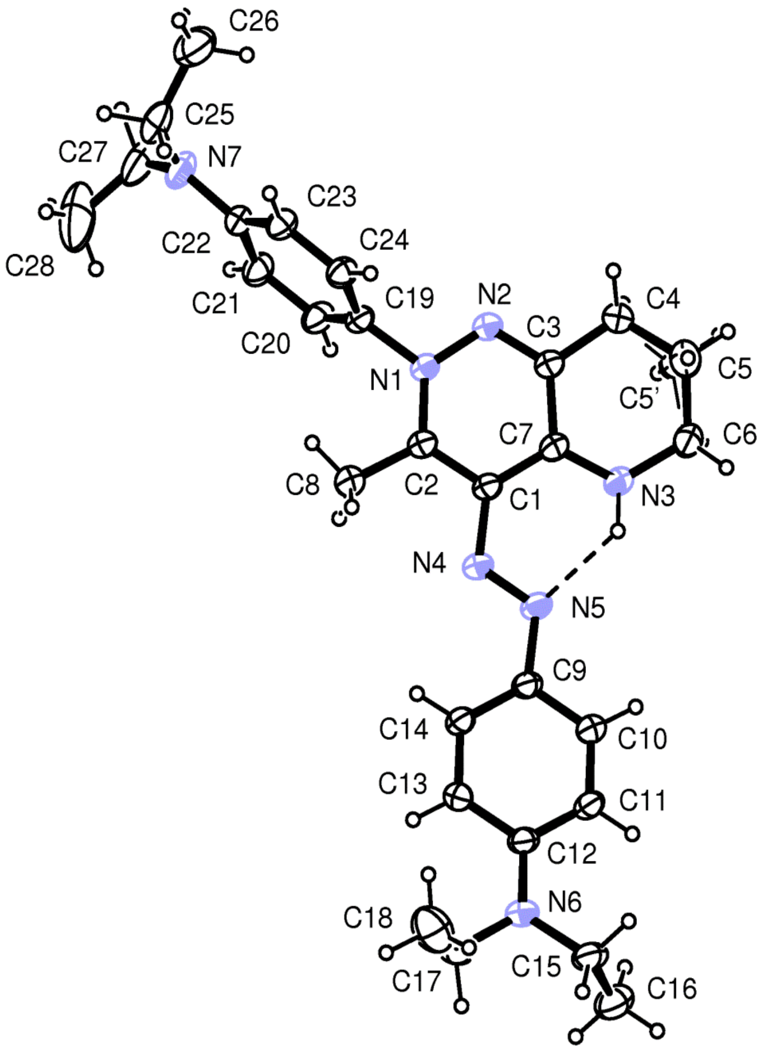

Figure S3: ORTEP view of the cation of compound **5d** showing the thermal ellipsoids at 30% probability level. Both the disordered $C5H_2$ and $C5'H_2$ moieties are displayed.

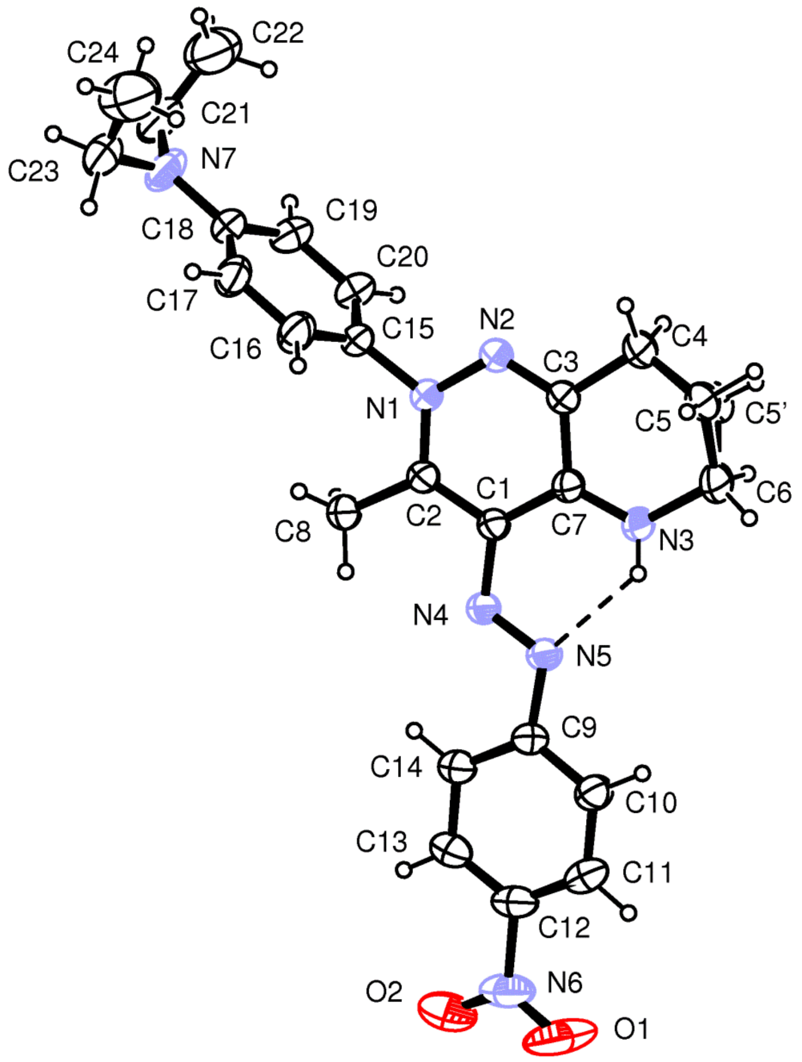

Figure S4: ORTEP view of the cation of compound **5f** showing the thermal ellipsoids at 30% probability level. Both the disordered $C5H_2$ and $C5'H_2$ moieties are displayed.

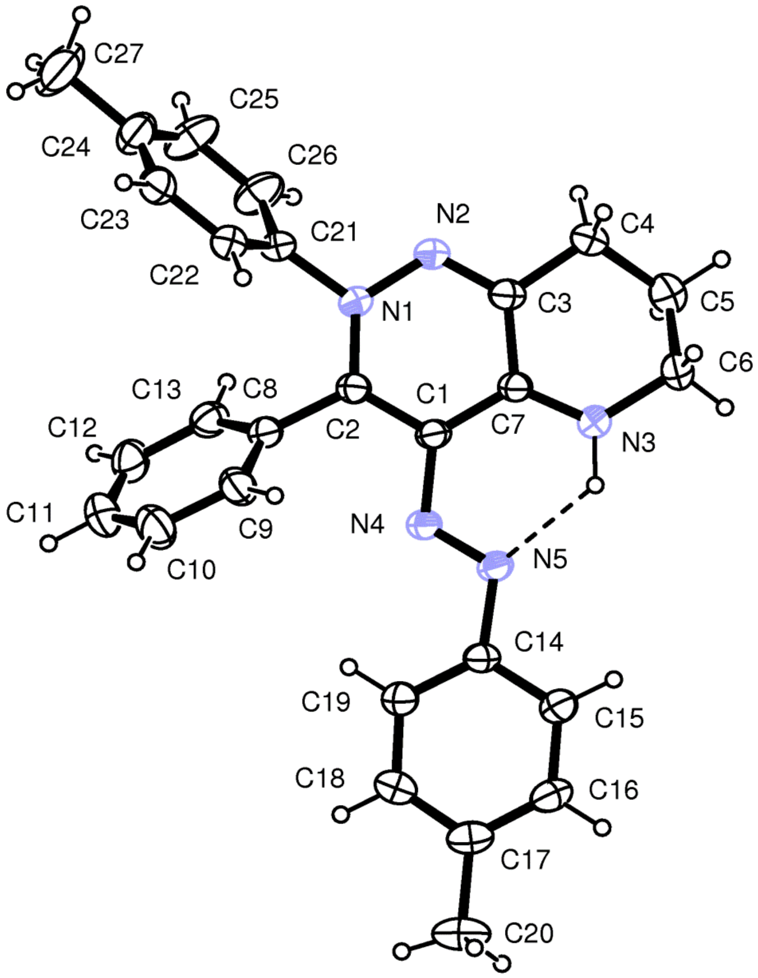

Figure S5: ORTEP view of the cation of compound **5g** showing the thermal ellipsoids at 30% probability level.

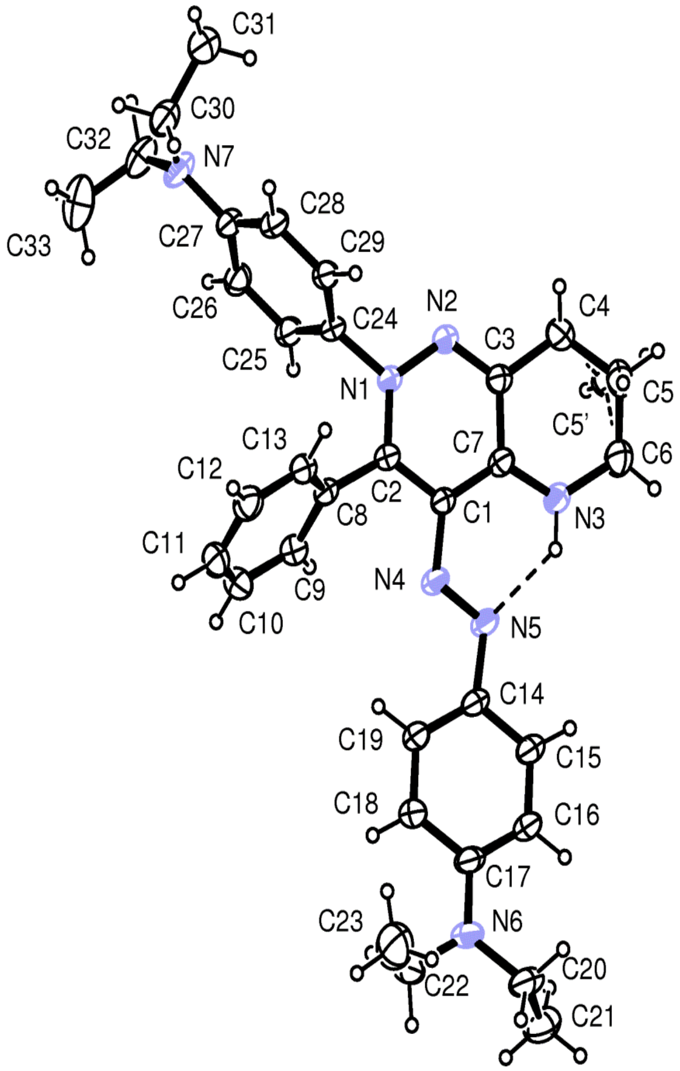

Figure S6: ORTEP view of the cation of compound **5j** showing the thermal ellipsoids at 30% probability level. Both the disordered $C5H_2$ and $C5'H_2$ moieties are displayed.

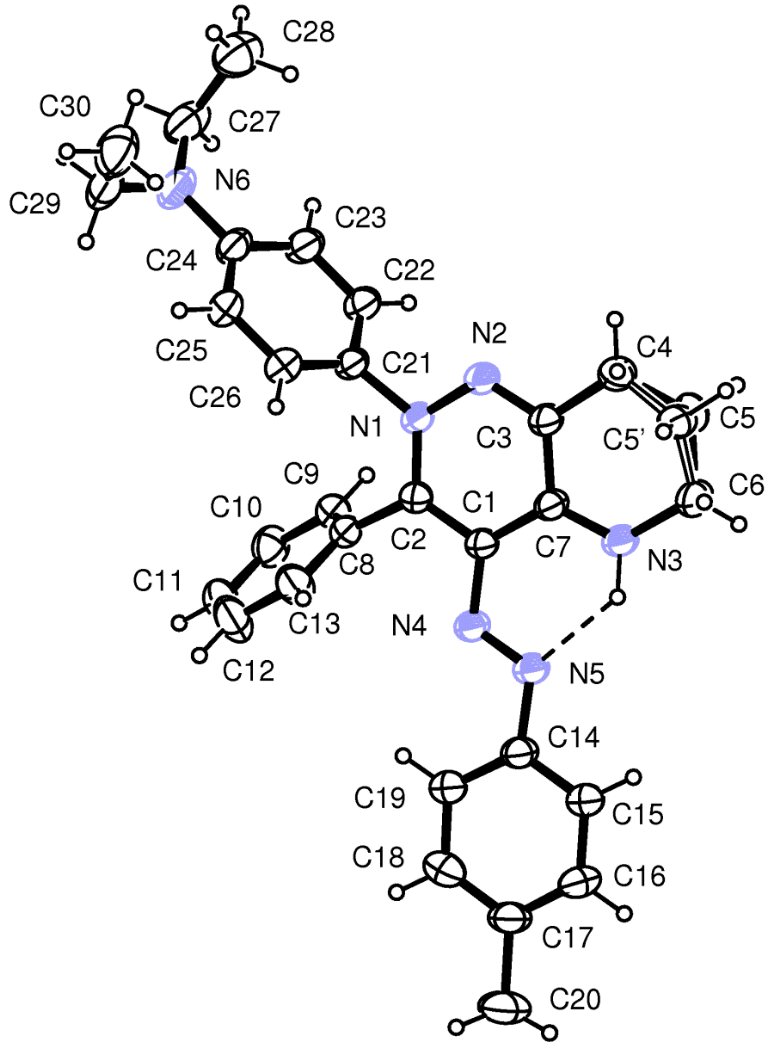

Figure S7: ORTEP view of the cation of compound **5l** showing the thermal ellipsoids at 30% probability level. Both the disordered $C5H_2$ and $C5'H_2$ moieties are displayed.

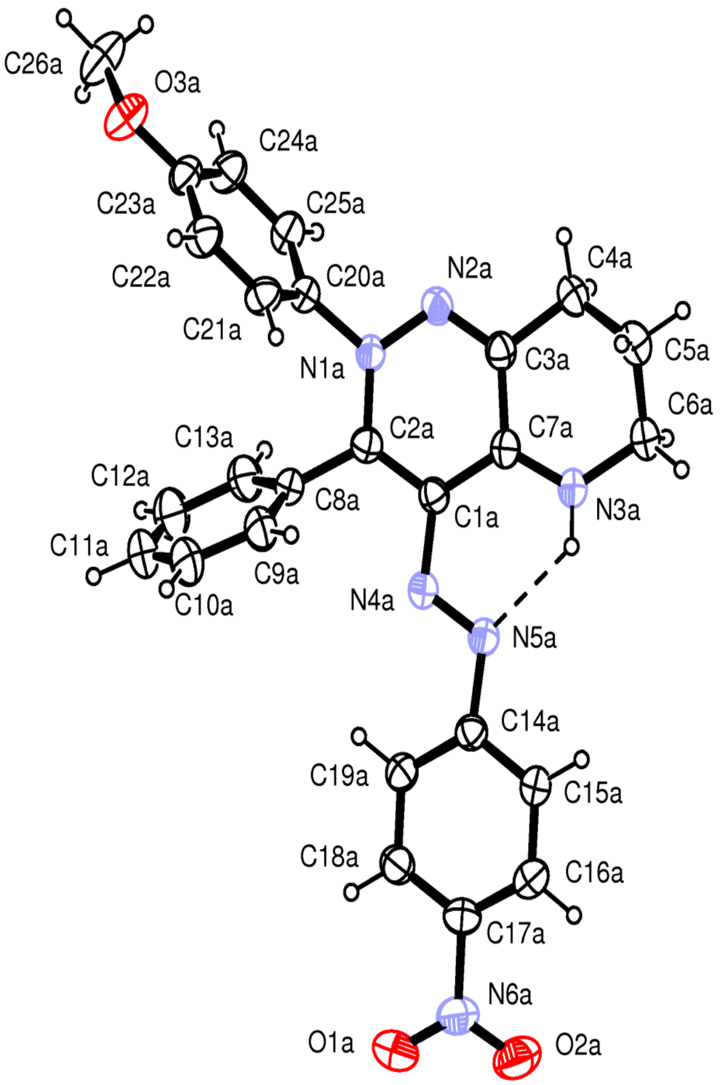

Figure S8: ORTEP view of the independent cation A of compound **5m** showing the thermal ellipsoids at 30% probability level. The two independent A and B cations differ only in the conformation of –OCH_3 substituent at the C20-C25 phenyl ring.

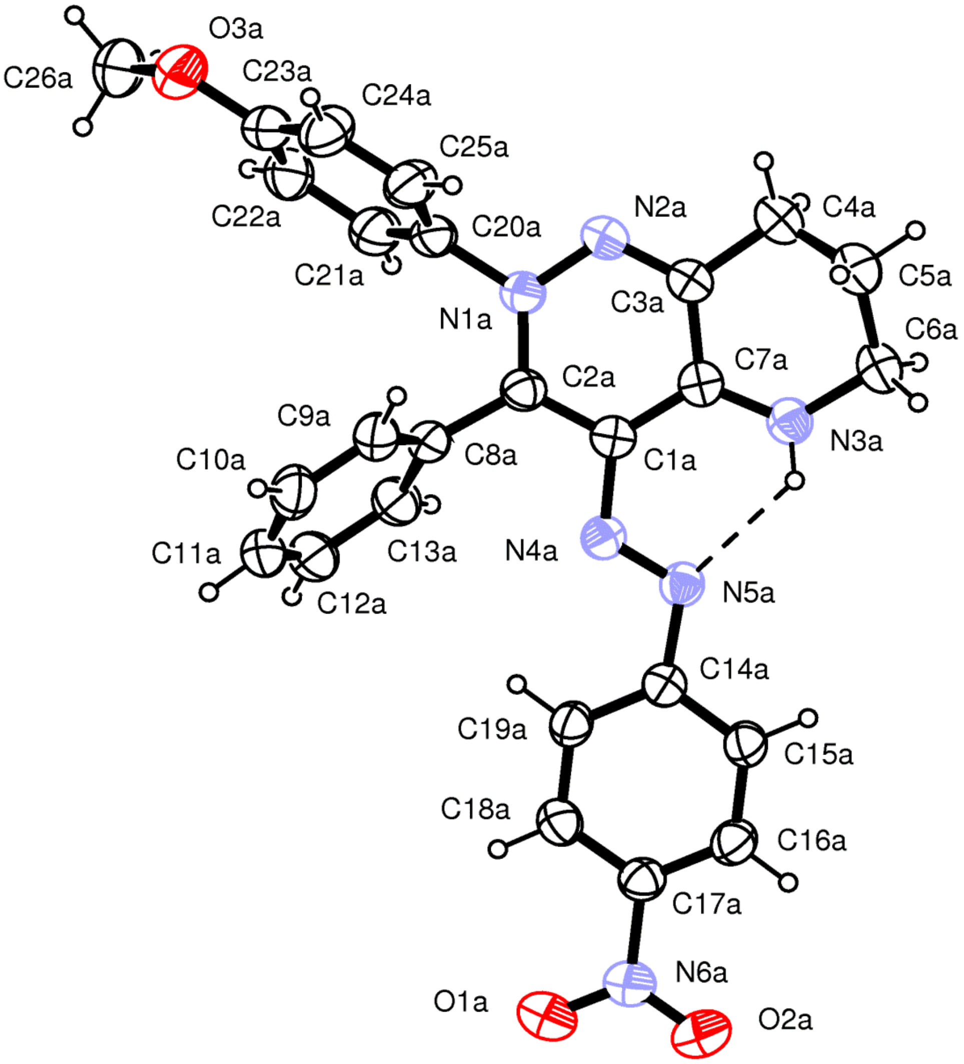

Figure S9: ORTEP view of the independent cation A of the mixture **5k + 5m** showing the thermal ellipsoids at 30% probability level.

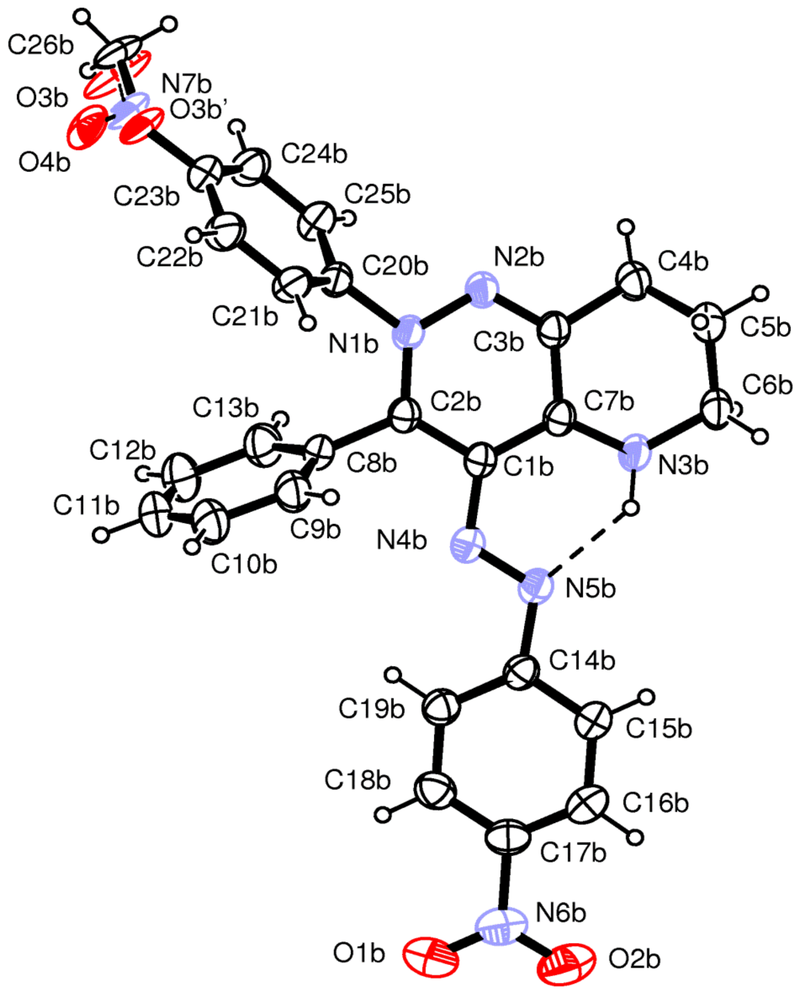

Figure S10: ORTEP view of the independent disordered cation B of the mixture **5k + 5m** showing the thermal ellipsoids at 30% probability level. Both the superimposed disordered –OCH_3 and -NO_2 *para*-substituents at the C20b-C25b phenyl ring are displayed .

References

1. Schann, S.; Bruban, V.; Pompermayer, K.; Feldman, J.; Pfeiffer, B.; Renard, P.; Schalbert, E.; Bousquet, P.; Ehrhardt, J.-D. *J. Med. Chem.* **2001,** *44,* 1588–1593. doi: 10.1021/jm001111b
2. Josefík, F.; Svobodová, M.; Bertolasi, V.; Šimůnek, P.; Macháček, V.; Almonasy, N.; Černošková, E. *J. Organomet. Chem.* **2012,** *699,* 75–81. doi: 10.1016/j.jorganchem.2011.11.004
3. Otwinowski, Z.;Minor, W. Methods in Enzymology, C.W. Carter, R.M. Sweet. Eds.; Vol. 276, Part A, Academic Press: London, U.K 1997, pp. 307–326.
4. Blessing, R. H. *Acta Crystallogr., Sect A* **1995,** *51,* 33–38. doi: 10.1107/S0108767394005726
5. Altomare, A.; Burla, M. C.; Camalli, M.; Cascarano, G. L.; Giacovazzo, C.; Guagliardi, A.; Moliterni, A. G.; Polidori G.; Spagna, R. *J. Appl. Crystallogr.* **1999,** *32,* 115–119. doi:10.1107/S0021889898007717
6. Sheldrick, G. M. SHELX-97, University of Göttingen, Germany, 1997.
7. Nardelli, M. *J. Appl. Crystallogr.* **1995,** *28,* 659–659. doi:10.1107/S0021889895007138
8. Spek, A.L. *Acta Crystallogr., Sect. D* **2009,** *65,* 148–155. doi: 10.1107/S090744490804362X
9. Farrugia, L. J. *J. Appl. Crystallogr.* **1999,** *32,* 837–838. doi:10.1107/S0021889899006020
